# Evolutive Models for the Geometry and Heat Conductivity of an Intumescent EVA-ATH Composite during Its Thermal Degradation

**DOI:** 10.3390/ma13225258

**Published:** 2020-11-20

**Authors:** Jianwei Shi, Germain Boyer, Valeri Mourzenko, Jean-François Thovert

**Affiliations:** 1 Institut P’, CNRS—Université de Poitiers—ISAE-ENSMA, 11 bd Marie et Pierre Curie, TSA 41123, CEDEX 09, 86073 Poitiers, France; jianwei.shi@univ-poitiers.fr (J.S.); mourzenko@ensma.fr (V.M.); 2 Institut de Radioprotection et de Sûreté Nucléaire (IRSN), PSN-RES/SA2I/LIE, Cadarache, 13115 St Paul Lez Durance, France; germain.boyer@irsn.fr

**Keywords:** intumescent polymer, composite, thermal degradation, morphology, thermal conductivity, conceptual modeling, tomography, upscaling

## Abstract

Reliable predictions from numerical simulations in fire safety applications require knowledge of the combustible materials’ properties in their initial and thermally degraded states. The thermal conductivity of the sheath material of electrical cables, present in massive amounts in industrial plants, is addressed here. An evolutive conceptual model is proposed for the morphology of this intumescent polymer composite during its thermal degradation. It accounts for the multiscale structure and anisotropy observed during a thorough characterization based on tomographic images of samples at representative stages of the degradation. The evolution of the geometrical characteristics during the process is linked to chemical advancement parameters according to a reasoned scenario based on physical arguments and balance considerations. The anisotropic thermal conductivity tensor can be deduced from the geometry by a nested application of classical models. Ultimately, the conductivity is obtained as an analytic function of the chemical advancement and temperature. The model predictions were validated by comparisons with direct numerical solutions of thermal problems in the fully described geometry provided by the tomographies, and with measurements from the literature. The methodology and conceptual tools can be of interest for the treatment of other materials and in other contexts of application.

## 1. Introduction

Fire spreading along cable trays in nuclear power plants has been widely studied during the past decade, demonstrating the influences of the cables’ arrangement and of the tray’s loading, spacing and width. However, recent large-scale experiments in the framework of the OECD PRISME program lead by the French Institute for Radiological Protection and Nuclear Safety (IRSN) showed that the cable-sheath materials also play a key role in the fire growth [1]. Pyrolysis models coupled with computational fire dynamic simulations [2,3,4,5] require knowledge of the thermal and thermo-kinetic properties of the sheath materials all along their degradation processes, for the correct prediction of the fire scenario. Reliable descriptions can be achieved for non-charring polymers. However, the prediction of the degradation rates of polymers which produce a porous residue stumbles by lack of correct accounts of the thermal properties—primarily the thermal conductivity, which cannot be easily characterized because of the geometrical evolution. Therefore, these parameters are generally adjusted by an optimization procedure [3,6]. In order to resolve this bottleneck, a sheath material composed of a polymer (EVA, ethylvinyl acetate) mixed with a fire retardant mineral filler (ATH, alumina trihydrate) is considered in the present work. A thorough characterization of its evolving morphology was conducted. Then, evolutive models for the material geometry, and in turn for its conductivity, were formulated. Note that other relevant effective coefficients such as the density or heat capacity were appropriately evaluated by a simple volume average of the constituent properties. For this reason, they are not addressed here. The attention is focused on the thermal conductivity, which cannot be estimated without accounting for the microstructure.

There is a long history of conductivity modeling in porous media, by analytical approaches for idealized structures or by numerical upscaling for complex random ones [7,8,9], but intumescent polymers have been addressed only relatively recently. The thermal conductivity of intumescent coating was considered in [10,11]. Analytical models for idealized geometries were applied and the importance of parameters such as the pore size distribution was discussed, but the geometry of the degrading material was observed only in a section of the material in its ultimate residual state. The 2D pore distribution of an intumescent polymer was measured by scanning electron microscopy (SEM) in [12]. The effective conductivity was evaluated by direct numerical simulation (DNS) in the 2D images with a subsequent conversion to 3D based on the assumptions of an isotropic structure and spherical pores. Most recently, careful geometrical measurements were conducted in 2D sections through degraded intumescent polymers and the porosity and pore size distribution were analyzed [6]. Establishing a quantitative relationship between the thermal transport properties and the char structure is presented as a future extension of this work. Finally, X-ray computed tomography was first used in [13] for a rich characterization of the geometrical and compositional features of an intumescent polymer at various stages of its degradation, and later in [14], wherein a reasoned scenario and a conceptualized model for the morphological evolution were proposed. However, the prediction of conductive properties from this geometrical knowledge was not addressed.

In a different approach, the development of porosity and intumescence is sometimes modeled a priori with the use of a bubble growth model [15,16]. While the idea of a physically based modeling is commendable, the method does not yet seem able to accommodate some of the multiscale and anisotropic features revealed by direct observation in the following.

From an opposite point of view, it is possible to directly measure the effective conductivity rather than modeling it from the material structure. This was done in [17,18] for an EVA-ATH composite, in its initial state and intermediate (after ATH dehydration) and final degraded states. Then, the conductivities of these pseudo-species can be combined with a mixing rule to deduce the effective conductivity of the material from its current composition (or equivalently from chemical advancement parameters) in any stage of its degradation. However, the geometric characteristics and therefore the conductivity are by no means linear functions of the advancement, as shown in the following, and such interpolations can result in significant errors.

In the present work, a thorough geometrical characterization of the degrading EVA-ATH composite is conducted first in Section 2, based on 2D SEM and 3D tomographic images in representative states, namely, after the successive completions of the ATH dehydration and of the EVA decomposition. This reveals a multiscale and anisotropic structure, which is conceptualized in Section 3 into a geometric model involving the volume fractions of pores on a cascade of scales and an aspect ratio for the largest ones. Furthermore, physical arguments and volume balance considerations are used to establish a reasoned scenario for the material’s morphological evolution all along its degradation process, and in turn for the evolutions of the model parameters as functions of chemical advancement.

This geometrical representation is combined in Section 4 with classical analytical results to obtain an evolutive thermal conductivity model. Its predictive capabilities are checked by comparison with the results of DNS performed in the 3D tomographic images. In view of the moderate sizes of the pores, radiative exchanges are expected to contribute only marginally to the heat transfers and they are not considered. The validity of this assumption is checked a posteriori in Section 5, where the sensitivity to some sources of errors is discussed and a comparison is made with measurements from the literature of the thermal conductivity of a similar composite in various states of degradation. Concluding remarks and perspectives for improvement or extension of the modeling approach are given in Section 6.

Note that this article summarizes part of the work described in [19]. For concision, only a limited set of illustrations is provided here and many technical points are mentioned without detailed descriptions. More extensive illustrations and a detailed account of all technical aspects and theoretical developments can be found in [19].

## 2. Geometric Characterization

### 2.1. Characteristics of the Investigated Material

Fire safety in industrial plants (and especially nuclear facilities) is one of the major concerns of IRSN. This involves optimizing the materials and the design of new installations, but also assessing the risks in existing ones, which often contain significant numbers of electric cables with combustible sheaths. Therefore, the investigated EVA/ATH sheath sample was taken from the most recent generation of cables used in existing and future plants. A fortunate circumstance is that EVA/ATH is also the only polymer composite for which we found direct measurements of the conductivity at different stages of its thermal degradation in the literature. This provides the reference for the comparison and discussion of the model predictions in Section 5.2.

Note that the ATH filler acts as a fire retardant, mostly through the intumescence it induces when it dehydrates which results in a thermal barrier effect. This a sought-after feature for fire safety and many polymer-based composites, including mineral fillers which play a similar role (see, e.g., [20]). Therefore, many composites are expected to present behaviors similar to the investigated one, and the concepts, techniques and modeling approaches presented here are potentially applicable to a wide class of materials.

The sheath is 3 mm thick and composed of polymerized ethylene and vinyl acetate (EVA), containing 60% by mass of a mineral filler, alumina trihydrate (ATH, chemical formula Al(OH)${}_{3}$), which acts as a flame retardant. When exposed to fire, the material undergoes a chemical degradation which can be schematized in two successive steps: first the dehydration of ATH (very endothermic, hence the fire retardant effect), and then the decomposition of EVA, which leaves no solid residue. According to the thermogravimetric analysis (TGA), these processes take place in non overlapping ranges of temperature. Hence, the material evolves from its initial state denoted by [S0] to an intermediate state denoted by [S1] where the polymer is still intact but ATH is entirely dehydrated into alumina, and to a final state [S2] where EVA is entirely decomposed. The final residue contains only alumina.

**Figure 1:**
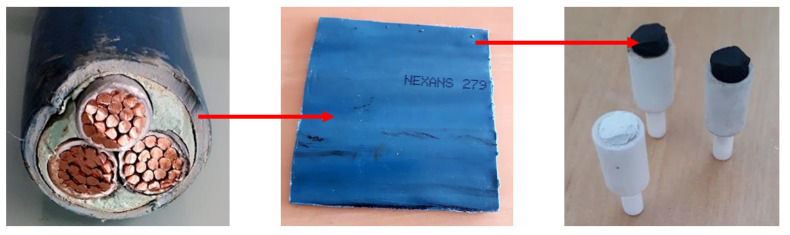
Sample preparation. The black samples in the right picture correspond to state [S1] and the white one to state [S2]. Note the intumescence in the degraded states (the samples in their initial states fitted in the crucibles).

Samples of degraded materials were prepared as detailed in [21], by placing cylindrical pieces cut from a flattened cable sheath in an oven at a temperature of about 400 ${}^{{}^{\circ}}$C for [S1] and 900 ${}^{{}^{\circ}}$C for [S2] (Figure 1). Then, the microstructures of these two samples were characterized by SEM and X-ray tomography, as detailed in the following.

### 2.2. SEM Observation

**Figure 2:**
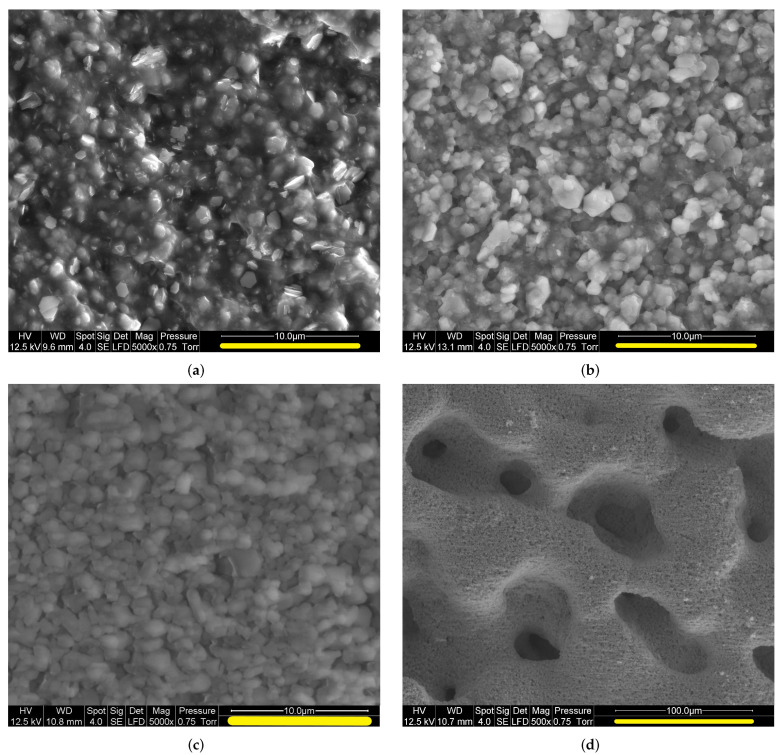
SEM observations of the outer surface of the material in the successive states [S0] (**a**), [S1] (**b**) and [S2] (**c**,**d**). The yellow streaks correspond to 10 μm in (**a**–**c**) and to 100 μm in (**d**).

SEM images of the outer surfaces of samples in successive stages, obtained at C2MA (Alès, France) [21], are shown in Figure 2. The mineral grains, composed of ATH in state [S0] and of alumina in states [S1] and [S2], are visible in Figure 2a–c. There is no evidence of evolution for their typical size, 2 μm, and shape, roughly spherical, during the pyrolysis. Therefore, it is assumed in the modeling process that these characteristics remain constant. The picture with a lesser magnification in Figure 2d for state [S2] shows the existence of a macroporosity, with channels and cavities in a range of sizes 10 to 100 μm. A thorough characterization of the macroporosity is described in Section 2.3, based on 3D tomographic images. Macropores also exist in state [S1], but they do not show up on the outer surface.

### 2.3. Tomography Observation

The X-ray tomographic images of the samples, obtained at LEM3 (Metz, France), resulted from complete scans of the sample with 991 rotations with respect to the *z*-axis. For [S1], the *z*-direction corresponds to the crucible axis, i.e., to the direction along which intumescence could take place. For [S2], only debris of the very brittle residue sample could be recovered and the orientation is unknown. The native voxels size was 3.3 μm. However, due to poor contrast of the grayscale images and intense noise, an elaborate pretreatment was required to clearly distinguish solid and gas phases ([19], Chap. 5 and App. A.1). This involved spatial filtering, and as a result, only supervoxels consisting of 3${}^{3}$ native voxels were kept in the working images. Their size of 10 μm corresponds to the effective spatial resolution. From this point on, the term “voxel” always refers to these supervoxels. Note that for practical reasons, illustrations are generally provided in 2D cross-sections, but all the treatments and characterizations have been carried out using 3D morphological operations.

**Figure 3:**
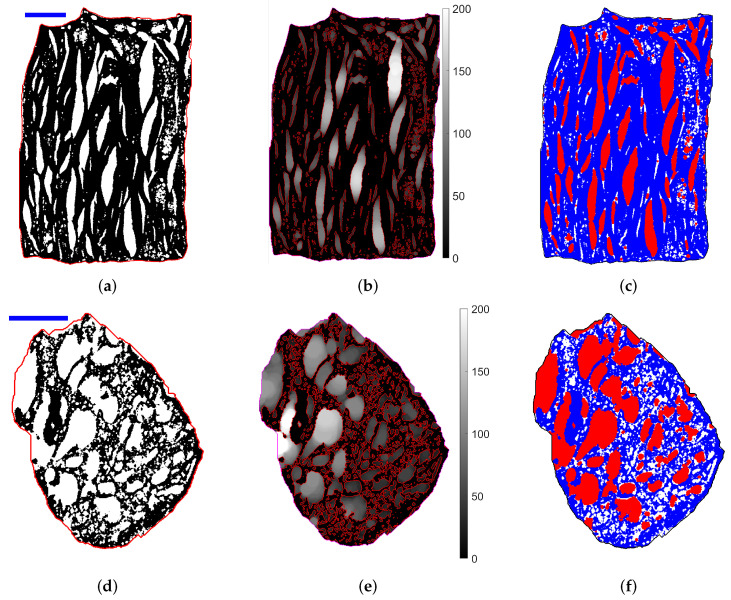
Cross-sections perpendicular to the *x*-axis in [S1] (top row) and [S2] (bottom row). The *z*-axis is horizontal. Thresholded tomography (**a**,**d**); the red line represents the outer sample envelope; the blue streaks correspond to 1 mm. Covering radius $R_{c}$ (μm) in the pores (**b**,**e**): the red lines indicate the contour of the solid phase. Mesopores (white), macropores (red) and apparent solid phase (blue) with a threshold $R_{c,s}$ = 30 μm (**c**,**f**).

Examples of sections through the [S1] and [S2] samples are shown in Figure 3a,d. The red contours are the sample envelopes obtained by a dilation/erosion algorithm, including the macropores open to the outside. Pores (white) were observed within an apparent solid phase (black). As shown in Section 2.4 on the basis of mass and volume balance considerations, this solid phase has to be microporous, on a scale smaller than the 10 μm resolution of the images. Thus, we call “apparent” what is visible in the tomographies, namely, apparent (microporous) solid and corresponding apparent porosity, with volume fractions $\Phi_{app}$ = 0.257 in [S1] and 0.519 in [S2].

Note that no macropore shows at the outer surface in [S1], although paths for the evacuation of the vapour released by the ATH dehydration must have existed. They were probably kept open by the exuding flow through the liquefied EVA but closed due to capillary forces during the cooling of the samples and the EVA solidification.

Intumescence takes place in the early stages of the degradation process of the EVA-ATH mixture, due to the trapping of gases in the molten EVA. It is reasonable to assume that the swelling stops when the development of porosity leads to the appearance of a continuous path for the gas evacuation. Visual observation during cone calorimeter tests confirmed this scenario, and also showed that no sagging occurs in the final phase. Therefore, the intumescence factor can be measured using the tomographic image for [S1]. The sample height in the crucible, 3 mm for [S0], increased to 4.2 mm. The intumescence factor is therefore β = 4.2/3 = 1.4. Note that this measurement is not very accurate due to the irregularity of the sample’s surface. Furthermore, we got images of the cooled degraded sample. At the typical temperature (∼350 ${}^{{}^{\circ}}$C) where state [S1] is reached, the density of EVA is probably slightly smaller and β slightly larger than after cooling. The sensitivity of the conductivity modeling to the value of β is studied in Section 5.1.

#### 2.3.1. Pore Size Spectrum and Scale Separation

**Figure 4:**
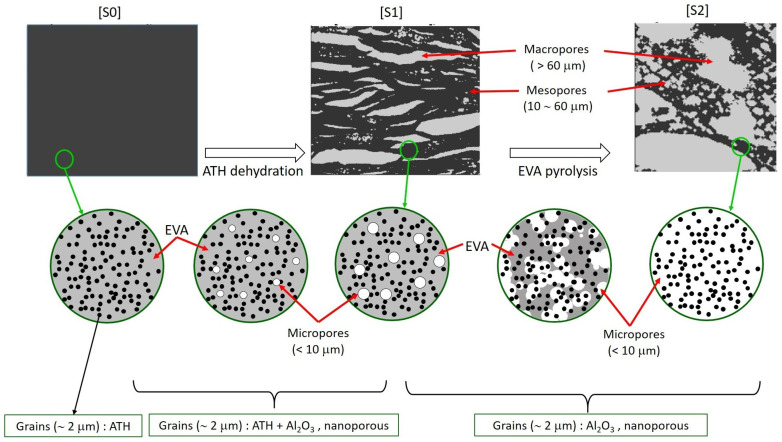
Multiscale structure of the material in its successive states [S0], [S1] and [S2] (left to right).

Figure 3a,d suggests a multiscale structure involving two populations of bubbles with separated typical sizes, which are referred to as meso and macro-bubbles. Recall that micropores unseen in the tomographies also exist in the apparent solid. This hierarchical structure is illustrated in Figure 4. Furthermore, the largest inclusions are very anisotropic, while the smaller ones are fairly spherical. This is consistent with an argument which predicts that bubbles in the molten polymer smaller than 100 μm cannot strongly depart from sphericity due to dominant capillary forces ([19], App. A.3).

The concept of covering radius $R_{c}$ [22] was used to provide a quantitative basis for the subsequent treatments. $R_{c}$ at a position r is defined as the radius of the largest sphere entirely contained in the pore space which can cover r. It essentially measures the “thickness” of the pore space in the vicinity of r. Examples of $R_{c}$ fields are given in Figure 3b,e. Since $R_{c}$ is determined in 3D, it can be smaller or larger than expected from the sole consideration of the 2D section.

The covering radius $R_{c}$ ranges from 10 to 200 μm (in [S1]) or 280 μm (in [S2]), and its spectrum actually extends below 10 μm if the unseen microporosity is taken into account. This spectrum is continuous and does not really comply with the visual perception of a modal distribution. Nevertheless, a schematized trimodal representation with micro, meso and macropores is adopted for simplicity in the modeling process. The separation between micro and mesopores is dictated by the 10 μm spatial resolution. The separation between meso and macroporosity is set at a threshold $R_{c,s}$. Since no self-evident value results from the $R_{c}$ spectrum, values of $R_{c,s}$ ranging from 20 to 40 μm have been tested and their plausibility has been assessed by considering their compatibility with spatial correlation measurements (see Section 2.3.2), onset of bubble asphericity and visual perception. The full modeling process was carried out with $R_{c,s}$ = 25 and 30 μm, and the conductivity predictions were found nearly insensitive to the threshold value in this range. $R_{c,s}$ = 30 μm is used from this point on. The corresponding macro, meso and apparent porosities $\Phi_{M}$, $\Phi_{m}$ and $\Phi_{app}$ are given in Table 1.

The resulting separation in meso and macroporosity is illustrated in Figure 3c,f. The agreement with visual perception is gratifying, except for two features. First, small white areas show at the tapering ends of elongated macropores (Figure 3c). This defect stems from the semi-local character of $R_{c}$: it measures the “thickness” of a pore around a point, considering its vicinity but not the possible connection of a narrow region to a wider one. However, the volume fraction of the disputed areas is very small. Secondly, some white areas observed in Figure 3f would intuitively be interpreted as macropores. This discordance is not a defect. It results from the presence of solid out of but close to the displayed cross-section.

#### 2.3.2. Spatial Correlation and Anisotropy Determination

Spatial correlation is a powerful tool to detect and quantify anisotropy and possibly a multiscale characteristic. For a binary phase function *Z* such as our binarized tomographies, with *Z* = 1 in the pores and 0 in the solid space, the two-point correlation function $R_{Z}\left(u\right)$ is the covariance of the phase function at two points separated by the lag u normalized by its variance:

(1) $$R_{Z}\left(u\right)=\frac{\langle \;\left(Z\left(r\right)-\langle Z\rangle \;\right)\;\left(Z\left(r+u\right)-\langle Z\rangle \;\right)\;\rangle }{\langle \;{\left(Z-\langle Z\rangle \;\right)}^{2}\;\rangle }$$

where the brackets 〈.〉 denote a volume average.

**Figure 5:**
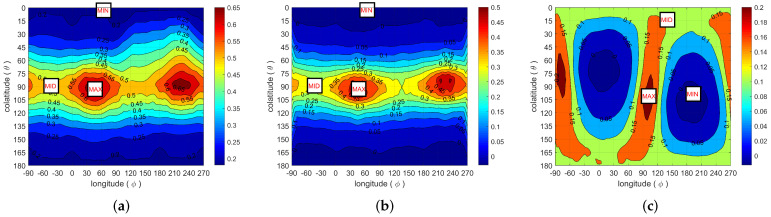
Spatial correlation $R_{Z}$ in [S1] at distances 8 (**a**) and 16 (**b**) voxels and in [S2] at a distance 16 (**c**). The labels MIN, MID and MAX indicate the eigendirections of the effective conductivity tensor.

The correlations measured with u on a sphere with radius 8 (for [S1]) or 16 (for [S1] and [S2]) voxel sizes are shown in Figure 5. Measurements at shorter or longer distances yield pictures similar to these examples. The direction of u is given by its polar angles ϕ (longitude) and θ (colatitude). Preferential directions are clearly visible. In [S1], a minimum takes place near the pole (θ≈ 0, *z*-direction), and a maximum (ϕ = 47${}^{{}^{\circ}}$) and a saddle point (ϕ = −43${}^{{}^{\circ}}$) are visible in the equatorial plane. These principal directions are associated with the anisotropy of the lenticular inclusions observed in Figure 3a, with short range correlations in the direction of their smallest dimension (*z*, horizontal in Figure 3a), and long range correlations in the (*x*, *y*) plane of their maximal extent. A similar pattern is observed for [S2], with a maximum and a minimum near the equatorial plane and a saddle point near the pole. Recall that whereas *z* is known to correspond to the crucible axis for [S1], the orientation of [S2] is unknown. It will be shown in Section 4.1 that the principal directions of the spatial correlation correspond also very accurately to the eigendirections of the thermal conductivity tensor.

**Figure 6:**
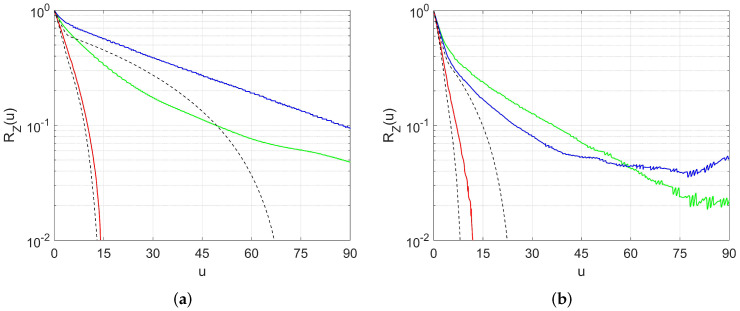
Spatial correlation $R_{Z}\left(u\right)$ in [S1] (**a**) and in [S2] (**b**), as a function of *u* in voxel-size units, when the lag u is set along the principal directions identified in Figure 5 (solid lines). The black broken lines are the correlations in a PEM/PSM sample, along the directions of the minor and major axes of the ellipsoids.

The spatial correlations measured along these directions are plotted as functions of distance in Figure 6. These plots reveal both the anisotropic and multiscale characters of the material. The curves for all directions start with steep and similar initial slopes, reflecting a quasi-isotropy at short distances. However, anisotropy develops at greater distances, with a transition to a slower decay for two of the curves. This behavior echoes the visual observations made in Figure 3. The initial slope of $R_{Z}$ is related to the volume density of pore/solid interface by

(2) $$2\Phi \;\left(1-\Phi \right)\;\frac{d}{du}\;R_{Z}{\left(up\right)}_{|u=0}=-\frac{A_{p}}{V}$$

where $A_{p}$ is the interfacial area projected along direction p in the volume V and Φ is the porosity. The density of interface results mainly from the large number of small pores, which do not show significant anisotropy. At greater distances, the correlation accounts for the strongly elongated large pores.

#### 2.3.3. Pore Segmentation and Equivalent Ellipsoids

For the purpose of morphological modeling, it is useful to supplement the global characterization in the foregoing, in terms of volume fractions and correlation functions, with statistics about the volumes and shapes of the constituting elements. The procedure described in Appendix A.1 makes use of the moments of inertia to characterize the shape of the individual pores. Ultimately, an equivalent ellipsoid is associated with each inclusion. The main features can be summarized as follows:

- The mesopores are fairly isotropic and they can be represented by spheres;
- The macropores are anisotropic, and they can be represented by oblate ellipsoids, with their minor semi-axis oriented along the direction corresponding to the most rapidly decreasing correlations.

These two kinds of objects are the basic constitutive elements of the conceptual geometrical model described in Section 3.1.

### 2.4. Apparent Solid, Nano and Micro-Porosity

As already noted, the apparent solid contains mineral grains and micropores finer than the tomography spatial resolution. It could not be examined by SEM observations either, because the degraded materials were too friable to allow their sectioning. Furthermore, nanoporosity also develops within the grains during their dehydration. These nano- and microscropic features have to be quantified by other means, based on global balances.

The dehydration of ATH, $2\mathrm{Al}{\left(\mathrm{OH}\right)}_{3}\;\to {\mathrm{Al}}_{2}O_{3}\;+\;3H_{2}O$, involves a mass loss for the mineral grains. However, the produced solid species is denser than ATH, with $\rho_{ATH}$ = 2400 kg/m${}^{3}$ and $\rho_{{Al}_{2}O_{3}}$ = 3950 kg/m${}^{3}$. Since the grain volume is regarded as constant, it implies that a porosity develops, called nanoporosity. The void fraction $\phi_{n}$ in the grains after complete dehydration ([S1], and of course [S2]) results from a mass balance of

(3) $$\phi_{n}^{\left(1\right)}=\phi_{n}^{\left(2\right)}=\;1-\frac{\rho_{ATH}}{\rho_{{Al}_{2}O_{3}}}\;\frac{W_{{Al}_{2}O_{3}}}{W_{ATH}}\;=\;0.603$$

where the superscripts ${}^{\left(0\right)}$, ${}^{\left(1\right)}$ and ${}^{\left(2\right)}$ correspond to states [S0], [S1] and [S2], respectively, and $W_{{Al}_{2}O_{3}}=102$ g/mol and $W_{ATH}=156$ g/mol are the molar mass of alumina and ATH.

The volume fraction $X_{grain}^{\left(0\right)}$ of grains in [S0] can be deduced from the initial 60% mass fraction and from the species intrinsic densities, $\rho_{EVA}$ = 900 kg/m${}^{3}$ and $\rho_{ATH}$ = 2400 kg/m${}^{3}$. This volume fraction evolves during the process, as a function of the intumescence factor β, with

(4) $$X_{grain}^{\left(0\right)}=0.375\;,\;X_{grain}=\frac{X_{grain}^{\left(0\right)}}{\beta }\;,\;X_{grain}^{\left(1\right)}=X_{grain}^{\left(2\right)}=0.268$$

Finally, the microporosity $\Phi_{\mu }$ undetected in the tomographies is evaluated as follows. The material [S1] contains mineral grains and EVA. The grain volume is constant, and since the tomographies are performed at room temperature, the density of EVA is identical for [S0] and [S1]. Thus, the intumescence corresponds to the pore volume fraction (nanoporosity within the grains excepted):

(5a) $$1+\beta \left(\Phi_{M}^{\left(1\right)}+\Phi_{m}^{\left(1\right)}+\Phi_{\mu }^{\left(1\right)}\right)=\beta \;,\;\Rightarrow \Phi_{\mu }^{\left(1\right)}=0.029$$

The same reasoning applies for [S2], but since only alumina remains, it reduces simply to

(5b) $$\Phi_{M}^{\left(2\right)}+\Phi_{m}^{\left(2\right)}+\Phi_{\mu }^{\left(2\right)}+X_{grain}^{\left(2\right)}=1\;,\;\Rightarrow \Phi_{\mu }^{\left(2\right)}=0.213$$

**Figure 7:**
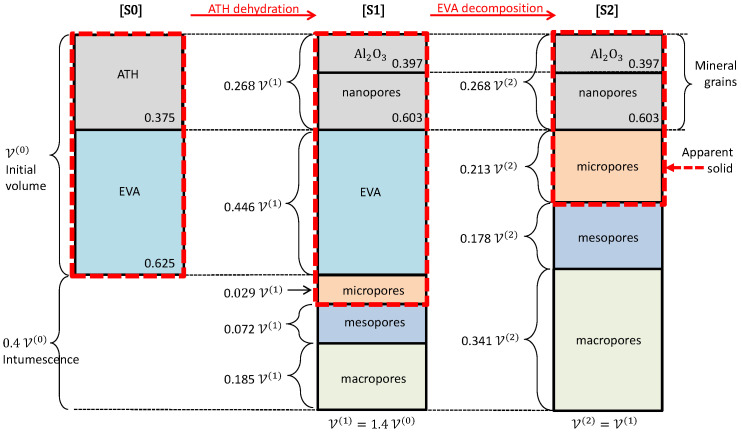
Volume fractions of the constituents and pores on various scales in states [S0], [S1] and [S2].

The volume fractions of all the constituents and pores on various scales in states [S0], [S1] and [S2] are summarized in Figure 7.

## 3. Evolutive Geometrical Model

A conceptual model is proposed here to represent the meso and macro-structure of the material in its successive states. Recall that we are interested in the thermal conductivity. Thus, the geometrical model does not have to account for every detail of the morphology, but only for the main features which primarily control its conductive properties. Its efficiency will be assessed in fine detail by its success in predicting the conductivity, in comparison with a direct numerical solution of the heat equation in the actual geometry described by the tomographies.

However, devising a geometrical model able to account only for the conductivity in states [S0], [S1] and [S2] is of limited interest, since it can be directly computed based on the available detailed digital images. A more ambitious and fruitful objective is to design the model so that it can represent the material in its continuous evolution from its initial to final states. In order to be operative, it should involve a sufficient but limited number of parameters, associated with a few simple conceptual elements. Furthermore, it is highly desirable that the conductivity can be predicted directly from the model characteristics, without need to resort to numerical simulations.

The model is introduced in Section 3.1 and its parameters are determined for states [S1] and [S2]. They all result from direct geometrical measurements, without any fitting or optimization procedure. Then, the evolutive model is presented in Section 3.2. The main task is the definition of the evolution laws for the parameters. Ideally, these laws should be determined from tomographies in a series of intermediate states. In the absence of such data, reasoned physical arguments are used to establish a scenario for the morphological evolution, and in turn for the evolution of the model parameters.

### 3.1. Pem/Psm—Morphological Model for [S1] and [S2]

The main lines of the modeling are to incorporate the general morphological features observed in the tomographies: spherical mesopores, and anisotropic macropores represented by ellipsoids. For simplicity, the void inclusions are randomly positioned, according to a Poisson process. Furthermore, a simplified bimodal distribution of the objects characteristics is used, with the same size for all individual spherical inclusions, and the same size, shape and orientation for the ellipsoidal ones. The reasons and justifications for this simplification are detailed in Appendix A.2. This does not preclude of course larger pores to appear, since the individual objects are free to overlap. Thus, the model is a combination of two Boolean models, PSM (penetrable sphere model) and PEM (penetrable ellipsoid model). It involves only two kinds of objects and five parameters, namely, two sizes, two volume fractions and an aspect ratio for the macropores.

The meso-inclusions are represented by spheres, with identical radii *R* = $R_{c,s}$ = 30 μm. The macro-inclusions are represented by parallel, monodisperse, biaxial oblate ellipsoids with semi-axes A×ηA×ηA, (η>1). The ellipsoid volume and aspect ratio are set equal to the volume-weighted averages of the measured values for the macro-inclusions, as detailed in Appendix A.2. The resulting minor axis *A* and aspect ratio η are given in Table 1.

Finally, the quantity of objects to be inserted is deduced from the measured porosities. For the macropores, the density $n_{E}$ of ellipsoids is related to their volume $V_{E}$ and the macroporosity $\Phi_{M}$ by

(6) $$\Phi_{M}=1-e^{-n_{E}\;V_{E}}$$

A similar formula applies for the spherical mesopores, involving their density $n_{s}$, volume $V_{S}$ and void fraction $\Phi_{m}/\left(1-\Phi_{M}\right)$ in the mesoporous material. When generating a stochastic digital sample from the model, meso and macro-porous solids are produced separately, with phase functions $Z_{m}$ and $Z_{M}$. The bimodal medium is then obtained by superimposing the $Z_{m}$ and $Z_{M}$ fields.

**Figure 8:**
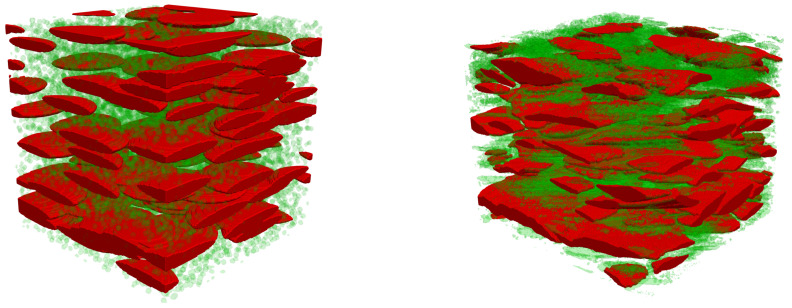
Illustration of the double-scale PSM/PEM reconstruction (**left**) and tomography (**right**) of the EVA-ATH material in the intermediate state of its pyrolysis. The green and red objects represent the meso and macroporosity, respectively.

For illustration, a sample generated using the PEM/PSM model for [S1] is compared in Figure 8 with the tomographic image. The overall structures are in good visual agreement. The correlation functions measured in reconstructed samples of [S1] and [S2], along directions parallel and perpendicular to the ellipsoids minor axis, are plotted in Figure 6 together with the correlations measured in the tomographies along the principal directions. The quantitative agreement is not perfect, but the model obviously captures the main features of the correlation properties. Note that no long range correlation (over distances larger than the object sizes) can be obtained with a Poissonian model. This does not mean that large pores cannot exist, since the individual objects can overlap and constitute large clusters, as seen in Figure 8. Besides, the correlations measured at very large distances (say more than 50 voxel sizes, or 500 μm) are not very reliable, because the statistical content of the digital images is less extensive, especially in the smaller [S2] sample.

### 3.2. A Scenario for the Morphological Evolution

An extension of the geometrical model is proposed here to represent the continuous morphological evolution of the material during its degradation. The basic conceptual elements are kept, supplemented with evolution laws for the number, sizes and shapes of the inclusions. Ideally, these laws should be determined from tomographies of the material in a series of intermediate states, but in the absence of such data, they are deduced from a reasoned scenario, volume balance considerations and physical arguments.

Let us first define two advancement parameters, which quantify the progresses of the two successive chemical processes, $\alpha_{1}$ for the ATH dehydration and $\alpha_{2}$ for the EVA decomposition.

(7) $$\alpha_{1}\;=\;\frac{m_{ATH}^{\left(0\right)}-m_{ATH}}{m_{ATH}^{\left(0\right)}}\;,\;\alpha_{2}\;=\;\frac{m_{EVA}^{\left(0\right)}-m_{EVA}}{m_{EVA}^{\left(0\right)}}$$

where $m_{ATH}$ and $m_{EVA}$ are the instantaneous masses of ATH and EVA while $m_{ATH}^{\left(0\right)}$ and $m_{EVA}^{\left(0\right)}$ are their initial values. The material evolves from states [S0] to [S1] and then to [S2] as $\alpha_{1}$ and $\alpha_{2}$ successively vary from 0 to 1.

The evolution of the porosities on the various scales has to be described first. The simplest aspect is the nanoporosity within the mineral grains. The dehydration leaves voids with a volume fraction which can be reasonably considered as proportional to the amount of dehydrated ATH, i.e.,

(8) $$\phi_{n}=\alpha_{1}\;\phi_{n}^{\left(1\right)}\;,\;\left(0\leq \alpha_{1}\leq 1\right)$$

where $\phi_{n}^{\left(1\right)}$ is the ultimate value (3) which applies for ($\alpha_{1}$=1, 0$\leq \alpha_{2}\leq$1). However, the complete dehydration, with a 35% mass loss [23], produces a huge quantity of steam and only a small amount can stay in the nanoporosity. The remaining is exuded in the surrounding molten EVA in the form of small bubbles. Recall that bubbles smaller than 100 μm cannot strongly depart from sphericity due to surface tension. In addition, the sedimentation velocity of these bubbles is negligible. Their rising speed can be evaluated by Stokes formula

(9) $$v_{asc}=\frac{2}{9}\;\frac{\Delta \rho \;g}{\mu }\;R^{2}$$

where *R* is the bubble radius, Δρ∼ 10${}^{3}$ kg/m${}^{3}$ the density difference between EVA and steam and μ the viscosity of the molten EVA. Data for μ in the literature are somewhat scattered, but a typical value μ∼10 Pa.s at the temperature of dehydration can be used for a rough estimate [24,25]. This yields $v_{asc}∼$ 0.02 μm/s if *R* = 10 μm and $v_{asc}∼$ 2 μm/s if *R* = 100 μm. Even in the latter case, a bubble would take about half an hour to travel the 3 mm thickness of the sample. Thus, the microbubbles remain in the EVA, causing the sample to swell (intumescence).

As their quantity increases, the microbubbles coalesce to produce larger ones, which constitute the mesoporosity, and in turn, macroporosity. As reported before, modeling the bubble size distribution in three categories is a simplified approach. The bubble size is limited for several reasons. First, it cannot exceed the thickness of the sample. Second, growing bubbles end up having a relatively fast rate of ascent. For example, $v_{asc}∼$ 0.2 mm/s if R∼ 1 mm. A bubble of this size could escape in no more than 15 s. Finally, as porosity increases, a continuous path appears through which the gas can escape. Then, an established regime takes place where the steam produced in the mineral grains is evacuated through a quasi-stationary multiscale pore system.

**Figure 9:**
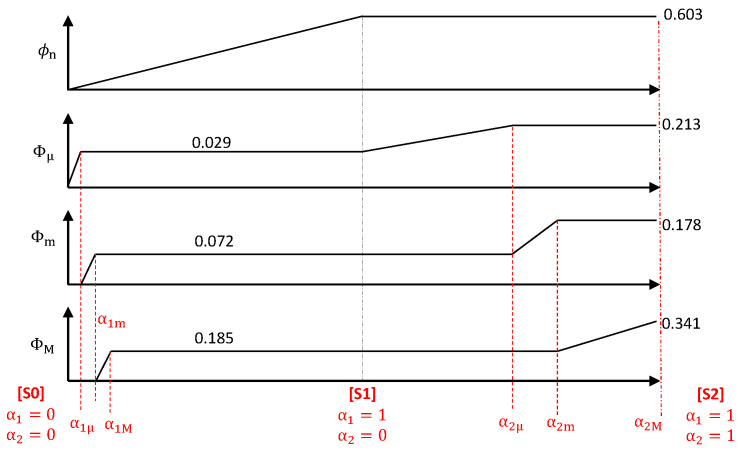
Evolution of the porosities on various scales as functions of the advancement parameters $\alpha_{1}$ and $\alpha_{2}$. $\phi_{n}$ is given with respect to the volume of mineral grains. $\Phi_{\mu }$, $\Phi_{m}$ and $\Phi_{M}$ are given with respect to the total sample volume. The threshold values of $\alpha_{1}$ and $\alpha_{2}$ are given in Table 2.

In summary, the modeling of the dehydration step rests on the following main ideas: In the first stage, steam is confined in the sample, which causes the development of the multiscale pore system and the intumescence; after a connected path to the outside appears, gases are evacuated, and the development of the pore system and the intumescence stop. Finally, since large bubbles necessarily result from the growth or coalescence of smaller ones, we consider in a schematized vision that the micro, meso and macroporosity develop sequentially, each of them up to the volume fractions observed in [S1]. The advancement $\alpha_{1}$ = $\alpha_{1\mu }$ when the produced amount of steam reaches the volume of the ultimate microporosity plus the corresponding nanoporosity, $\Phi_{\mu }^{\left(1\right)}+\alpha_{1\mu }\phi_{n}^{\left(1\right)}X_{grain}$, is easily determined. Similarly, $\alpha_{1m}$ and $\alpha_{1M}$ correspond to the advancements when the volumes of steam required to fill $\Phi_{m}^{\left(1\right)}+\alpha_{1m}\phi_{n}^{\left(1\right)}X_{grain}$ and $\Phi_{M}^{\left(1\right)}+\alpha_{1M}\phi_{n}^{\left(1\right)}X_{grain}$, respectively, have been produced. The values of these thresholds are given in Table 2. The evolution of the various void fractions is illustrated in the left part of Figure 9.

A similar approach is applied in the subsequent step, during the decomposition of EVA which produces pyrolytic gases without solid residue, causing the porosity to increase further by taking over the volume initially occupied by EVA. Microbubbles appear by nucleation in the molten polymer, which can coalesce into larger ones. The sequential character of the increases of the micro, meso and macroporosity is less univocally justified than during the dehydration since large bubbles can grow due to EVA decomposition at their surface in addition to the coalescence with smaller ones. Nevertheless, we keep this schematic sequential scenario, due to lack of knowledge for devising a more elaborate model. The increment of the total porosity from [S1] to [S2] is spread as a linear function of $\alpha_{2}$, $\Phi_{\mu }$, $\Phi_{m}$ and $\Phi_{M}$ reaching successively their ultimate values at advancements $\alpha_{2\mu }$, $\alpha_{2m}$ and $\alpha_{2M}$. These thresholds are given in Table 2 and the evolution of the void fractions is illustrated in the right part of Figure 9.

Finally, the characteristics of the inclusions in the evolutive PEM/PSM model have to be determined. The size parameters *A* for the ellipsoids and *R* for the spheres actually play no role in the final conductivity model (see Section 4.3). Only their volume fractions matter, and they have been described in the above. Thus, the last remaining point is the evolution of the aspect ratio η of the ellipsoids, which is different in states [S1] and [S2]. In the absence of explicit information, the simplest approach is to assume that the aspect ratio for the macropores varies between the known states [S0], [S1] and [S2] proportionally to their volume fractions,

(10a) $$\begin{array}{ccc} \frac{\eta -\eta^{\left(0\right)}}{\eta^{\left(1\right)}-\eta^{\left(0\right)}} & =\frac{\Phi_{M}}{\Phi_{M}^{\left(1\right)}}\;,\; & \mathrm{if}\;\;0\leq \Phi_{M}\leq \Phi_{M}^{\left(1\right)} \end{array}$$

(10b) $$\begin{array}{ccc} \frac{\eta -\eta^{\left(1\right)}}{\eta^{\left(2\right)}-\eta^{\left(1\right)}} & =\frac{\Phi_{M}-\Phi_{M}^{\left(1\right)}}{\Phi_{M}^{\left(2\right)}-\Phi_{M}^{\left(1\right)}}\;,\; & \mathrm{if}\;\;\Phi_{M}^{\left(1\right)}\leq \;\Phi_{M}\;\leq \;\Phi_{M}^{\left(2\right)} \end{array}$$

**Figure A1:**
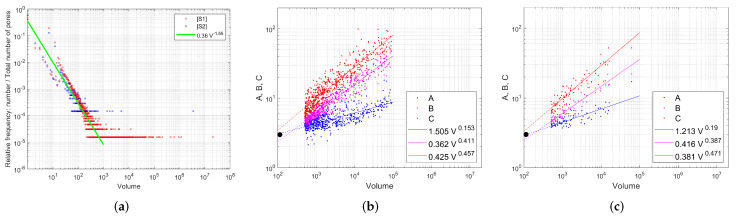
Distribution of the individual pore volumes in [S1] (red) and [S2] (blue) (**a**). Semi-axes of macropores deduced from the moments of inertia as functions of the pore volume, in [S1] (**b**) and [S2] (**c**). All distances are in voxel-size units. The black dots in (**b**,**c**) correspond to spheres with radius $R_{c,s}$.

The values of $\Phi_{M}^{\left(1\right)}$, $\Phi_{M}^{\left(2\right)}$, $\eta^{\left(1\right)}$ and $\eta^{\left(2\right)}$ are given in Table 1. In addition, $\eta^{\left(0\right)}$ is taken to be equal to 1, on account of the observation that the inclusions’ anisotropy increases with their size (see Figure A1), and that the first bubbles to appear are necessarily small.

## 4. Thermal Conductivity Modeling

In this section, the thermal conductivity of the material is evaluated first in states [S1] and [S2] by a direct numerical solution (DNS) of the heat equation based on the detailed image of their actual geometry provided by the tomographies. The effective conductivity tensor Λ is determined as a function of the conductivities $\lambda_{g}$ of the gas filling the pores and $\lambda_{s}$ of the apparent solid, which both vary during the degradation process due to changes of temperature and of the composition of these phases. This first result is then used as a reference to assess the capability of the PEM/PSM geometrical model to account for the thermal properties of [S1] and [S2], by comparing the conductivity predictions resulting from DNS in stochastically generated PEM/PSM samples to those obtained from the tomographies. In a third step, classical models are combined and applied to predict the conductivity tensor directly from the geometrical parameters, and the results are again compared to the DNS results. Finally, taking into account the evolution laws identified in Section 3.2, a fully analytical model is obtained which predicts Λ as a function of temperature and of the advancement parameters $\alpha_{1}$ and $\alpha_{2}$.

### 4.1. DNS Based on the Tomographies of [S1] and [S2]

Stationary thermal conduction in a heterogeneous medium with position-dependent thermal conductivity λ is governed on the local scale by Fourier’s law and a conservation equation:

(11) q=−λ∇T,∇·q=0

where q is the heat flux and *T* the temperature. However, if the medium statistical properties are uniform, it can be regarded on a larger scale as an equivalent homogeneous material with effective properties, including an effective conductivity Λ relating the locally averaged flux and gradient

(12) 〈q〉=−Λ·〈∇T〉,∇·〈q〉=0

Even though the local conductivity is assumed to be isotropic, quantified by scalars $\lambda_{g}$ for the gas and $\lambda_{s}$ for the apparent solid, the effective coefficient Λ is in general tensorial, since the medium structure can be anisotropic. For the materials under investigation and in the practical range of temperature, the local conductivity contrast satisfies 1 $\leq \lambda_{s}/\lambda_{g}\leq$ 20. However, a much wider range was explored, in order to test the limits of the conceptual model.

Equations (11) have to be supplemented with conditions at the boundaries of the computational domain. Their choice is not obvious, and it can affect the predicted effective tensor. A detailed study of the impact of various kinds of boundary conditions on the numerical solution was conducted and presented in [26]. In short words, in the practical range of local conductivity contrast, the predictions based on the periodicity conditions applied for the results presented hereafter cannot differ by more than 2% from those that would result from other reasonable choices of boundary condition in [S1]. Larger deviations up to 6% can arise in [S2] because of its smaller volume compared to its characteristic textural scale. These deviations are measured in terms of the tensorial distance defined in (A4).

The solver for the DNS is an improved descendant of that presented in [27], where the formulation is described in full details. Equations (11) are discretized in a finite volume formulation. Three computations are conducted for 〈∇T〉 set successively along the *x*-, *y* and *z*-directions. Λ is directly deduced via (12) from the three resulting mean fluxes 〈q〉.

As the solver can only operate in parallelepipedic domains, it was necessary to extract such blocks from the samples which have an approximately cylindrical ([S1]) or very irregular shape ([S2]). Their choice resulted from a compromise, aiming to maximize their volume while keeping their shape as close to cubic as possible. This resulted in blocks of [381 × 401 × 267] voxels for [S1] and [68 × 141 × 148] voxels for [S2]. In order to be representative, the block size should significantly exceed the scales associated with the microstructure. This can be assessed in terms of the semi-axes of the ellipsoidal macropores, with *A* = 6.6, *B* = *C* = 42.4 ([S1]) or *A* = 5.2, *B* = *C* = 14.4 ([S2]). The minor-axis is found along the *z* direction in [S1] and close to the *x* direction in [S2]. Hence, the block sizes in these terms are $\left[9\;C\times 9\;C\times 40\;A\right]$ for [S1] and $\left[13\;A\times 10\;C\times 10\;C\right]$ for [S2]. The volume of [S2] $\approx 1300\;AC^{2}$ is significantly smaller than that of [S1] $\approx 3240\;AC^{2}$. Thus, while both blocks have acceptable sizes, the statistical content of [S1] is larger than that of [S2].

Note that the values of *A* and *C* mentioned in the above are measured in the blocks and slightly differ from those in Table 1 measured over the whole samples, especially for [S1]. The major axis and the aspect ratio are slightly larger, which can be understood since the transverse development of the macropores is hindered near the crucible wall, whereas this region is excluded from the extracted block. In Section 4.2 and Section 4.3, the geometrical parameters measured in the blocks are used in PEM/PSM for meaningful comparisons with the DNS results in the real geometry and a correct assessment of the model performances.

Since the material is anisotropic, it is natural to analyze Λ in terms of its eigenvalues and eigendirections. For both [S1] and [S2], the eigendirections are nearly independent of the ratio $\lambda_{s}/\lambda_{g}$. They are shown in Figure 5 and found to coincide with the principal directions of the correlation function $R_{Z}$. In [S1], the smallest eigenvalue $\Lambda_{min}$ is associated with a direction very close to the *z*-axis, i.e., the direction of fastest decay of $R_{Z}$ and of the minor axis of the ellipsoidal macropores. The two other eigendirections associated with $\Lambda_{max}$ and $\Lambda_{mid}$ are in the equatorial plane in Figure 5a–c, along the same directions as the maximum and saddle of the correlation function. Similar features are observed for [S2], with eigendirections matching the principal directions of the correlation, as shown in Figure 5d–f. In this case, $\Lambda_{min}$ is found close to the *x*-axis, which is again the direction of fastest decay of $R_{Z}$ and of the minor semi-axis of the ellipsoidal macropores. Recall that the *z* direction is known to coincide in [S1] with the only possible direction of intumescence, which is the crucible axis. The orientation of [S2] is unknown. We assume that the direction of minimum correlation and conductivity corresponds, as in [S1], to the direction of intumescence.

**Figure 10:**
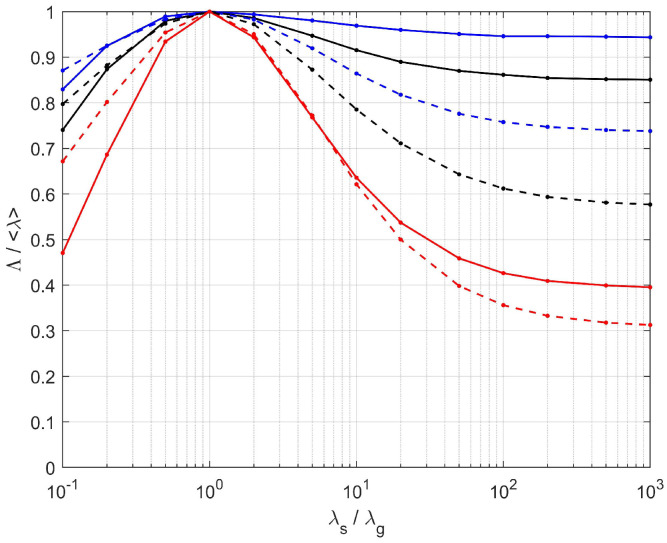
Eigenvalues of Λ obtained by DNS in the tomographies of [S1] (solid lines) and [S2] (broken lines), normalized by 〈λ〉, as functions of $\lambda_{s}/\lambda_{g}$.

The eigenvalues of Λ are plotted as functions of the ratio $\lambda_{s}/\lambda_{g}$ in Figure 10. For an easier comparison, they are normalized by the volume-averaged conductivity of the constituents $\langle \lambda \rangle =\Phi_{app}\lambda_{g}+\left(1-\Phi_{app}\right)\lambda_{s}$. Significant anisotropy develops in both [S1] and [S2] when the contrast increases, with $\Lambda_{max}/\Lambda_{min}$ of the order of 2 when $\lambda_{s}/\lambda_{g}\gg$1. It should be noted that the rough estimate 〈λ〉 of the effective conductivity which is used in many modeling approaches is a strong overestimation. This is especially true for $\Lambda_{min}$, and particularly concerning since it corresponds to the direction of intumescence. Therefore, it controls the heat transfer between the bulk of the material and its surface exposed to the fire, and should be modeled with particular care. Transfers in the transverse directions are of a smaller magnitude and they are disregarded in 1D models.

### 4.2. DNS in Pem/Psm Samples

Spatially periodic samples have been generated according to the PEM/PSM model, with the parameters associated with the blocks of [S1] and [S2]. The sample size was taken proportional to that of the ellipsoidal macropores, namely [20A× 20C× 20*C*]. It was checked that such a volume (much larger than that of the blocks extracted from the tomographies, see Section 4.1) prevents any systematic size effect and reduces statistical fluctuations sufficiently so that a single stochastic realization needs to be considered. The DNS were conducted using the same solver and over the same range of contrast $\lambda_{s}/\lambda_{g}$ as for the calculations in the tomographic images.

As expected, the eigendirections of Λ coincide accurately with the orientation of the ellipsoids, with $\Lambda_{min}$ in the direction of their minor axis. The two other eigenvalues are equal, since the macropores are represented by biaxial ellipsoids.

A thorough comparison of the conductivity tensors resulting from DNS in the tomographies and in the PEM/PSM samples is given in [19], in terms of the tensorial distance $D^{\prime }$ (A4). However, a simpler mode of comparison is used in the following, for two reasons. First, $D^{\prime }$ does not pay specific attention to the strategically most important direction associated with heat transfers from or to the surface exposed to fire, and since it corresponds to $\Lambda_{min}$, $D^{\prime }$ even puts more weight on relative errors of Λ along the less important other directions. Secondly, we argued that the departure from cylindrical symmetry in the real samples is incidental. It could be straightforwardly included in PEM by using triaxial ellipsoids if we regarded it as a meaningful feature, but since we did not, it should also be disregarded in the comparison with PEM. Therefore, since the tensors obtained from the tomographies and from PEM/PSM have the same structure with coinciding eigendirections, their difference is characterized by two scalars, namely $\Lambda^{\|}=\left(\Lambda_{mid}+\Lambda_{max}\right)/2$ in the planes parallel to the exposed surface and $\Lambda^{⊥}=\Lambda_{min}$ in the orthogonal direction, along which intumescence develops.

**Figure 11:**
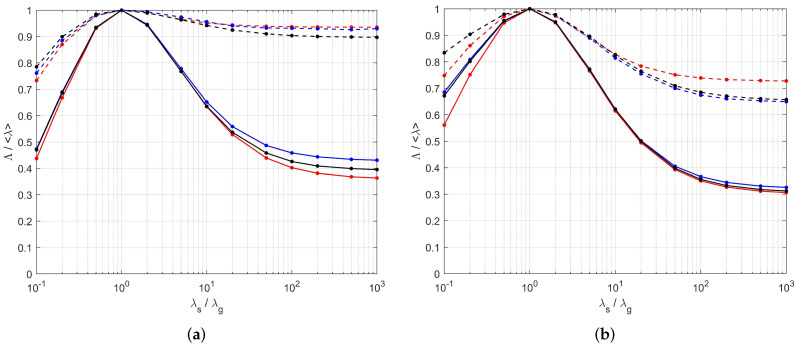
Components $\Lambda^{⊥}$ (solid lines) and $\Lambda^{\|}$ (broken lines) of Λ obtained by DNS in the tomographic images (black), by DNS in the PEM/PSM samples (blue) or by the analytic DEM model (17) (red), as functions of the contrasts $\lambda_{s}/\lambda_{g}$. Data in (**a**) and (**b**) correspond to [S1] and [S2], respectively.

These quantities, as obtained by DNS in the tomographic images and in the PEM/PSM samples for [S1] and [S2], are plotted in Figure 11 as functions of $\lambda_{s}/\lambda_{g}$. A good agreement is observed, especially in the practical range 1$\leq \lambda_{s}/\lambda_{g}\leq$20. A quantitative comparison is provided in Table 3, for various values of $\lambda_{s}/\lambda_{g}$. The most important case for practical applications is $\lambda_{s}/\lambda_{g}$=20, since it is the largest contrast expected with the materials under consideration. PEM/PSM is seen to account fairly well for the conductive properties, since its predicted heat fluxes deviate by at most 4% ([S1]) or 1.4% ([S2]), whatever the orientation of ∇T, from the reference provided by the tomographies. The deviations increase for much larger contrast, or in the unphysical case of $\lambda_{s}<\lambda_{g}$, but remain smaller than 9% even in the extreme case of $\lambda_{s}/\lambda_{g}$ = 10${}^{3}$.

### 4.3. Differential Effective-Medium Model (DEM)

In the vision of the structure identified in Section 2 and used in the PEM/PSM geometrical model, the pore space consists of inclusions (bubbles) in a matrix constituted by the apparent solid. A classical model from the literature is well suited to such situations, and it can be applied to predict the thermal conductivity directly from the geometrical parameters, without resorting to DNS calculations.

Consider first a matrix material with conductivity $\lambda_{1}$, containing spherical inclusions of another material with conductivity $\lambda_{2}$ and volume fraction $X_{2}$. In the differential effective-medium approximation (DEM) introduced by Bruggeman [28], the derivative dΛ/d$X_{2}$ of the effective conductivity Λ is evaluated by applying Maxwell formula [29] to quantify the effect of a small (i.e., dilute) additional amount of inclusions in an effective medium with conductivity Λ. The resulting differential equation integrated from $X_{2}$ = 0 yields

(13) $$\left(\frac{\lambda_{2}-\Lambda }{\lambda_{2}-\lambda_{1}}\right){\left(\frac{\lambda_{1}}{\Lambda }\right)}^{1/3}\;=\;1-X_{2}$$

The approach can be generalized to accommodate non spherical inclusions [8]. This involves the symmetric depolarization tensor $A^{*}$ associated with the inclusion shape. In the case of oblate biaxial ellipsoidal inclusions with semi-axes $a_{1}$ = $a_{2}$ = $\eta a_{3}$ (η≥1) and their minor-axis along the *z*-direction,

(14) $$A^{*}=\left[\begin{array}{ccc} Q & 0 & 0\\ 0 & Q & 0\\ 0 & 0 & 1-2Q \end{array}\right]\;,\;\mathrm{with}\;Q=\frac{1}{2}\left\{1+\frac{\eta^{2}}{1-\eta^{2}}\left[1-\frac{\tan^{-1}\left(\sqrt{\eta^{2}-1}\right)}{\sqrt{\eta^{2}-1}}\right]\right\}$$

If all the inclusions have identical orientation, the DEM argument can be repeated with a generalized Maxwell formula for ellipsoids. The effective tensor Λ has the same shape as $A^{*}$ and its diagonal components are given by

(15) $$\left(\frac{\lambda_{2}-\Lambda_{ii}}{\lambda_{2}-\lambda_{1}}\right){\left(\frac{\lambda_{1}}{\Lambda_{ii}}\right)}^{A_{ii}^{*}}=1-X_{2}$$

Note that for spheres, *Q* = 1/3 and (15) reduces to (13).

Let $\Psi_{ii}$ denote the solution of (15) for $\Lambda_{ii}/\lambda_{1}$. It is a function of the ratio $\lambda_{2}/\lambda_{1}$, of the volume fraction $X_{2}$ and of the depolarization coefficient $A_{ii}^{*}$. More specifically, if the inclusions are oblate biaxial ellipsoids, let $\Psi^{⊥}\left(\lambda_{2}/\lambda_{1},X_{2},\eta \right)$ be the solution for the direction of the minor axis ($A_{ii}^{*}=1-2Q$) and $\Psi^{\|}\left(\lambda_{2}/\lambda_{1},X_{2},\eta \right)$ the solution for the transverse plane ($A_{ii}^{*}=Q$). If the inclusions are spherical, Λ is isotropic and the superscript of Ψ can be dropped, $\Lambda /\lambda_{1}=\Psi \left(\lambda_{2}/\lambda_{1},X_{2}\right)$, solution of (13).

These elements can be combined and applied to the double scale structure of [S1] and [S2]. Assuming that the contrast of the meso and macropores’ sizes is sufficient, the ellipsoidal macropores can be considered as sitting in an equivalent homogeneous material representing the mesoporous material. The latter contains a volume fraction $\Phi_{m}/\left(1-\Phi_{M}\right)$ of spherical mesopores with conductivity $\lambda_{g}$ in the apparent solid with conductivity $\lambda_{s}$. Therefore, its conductivity is

(16) $$\Lambda_{meso}=\lambda_{s}\;\Psi \left(\frac{\lambda_{g}}{\lambda_{s}},\frac{\Phi_{m}}{1-\Phi_{M}}\right)$$

Then, a volume fraction $\Phi_{M}$ of ellipsoidal macropores with conductivity $\lambda_{g}$ sits in this material with conductivity $\Lambda_{meso}$. Consequently,

(17) $$\begin{array}{c} \Lambda^{\|}=\lambda_{s}\;\Psi \left(\frac{\lambda_{g}}{\lambda_{s}},\frac{\Phi_{m}}{1-\Phi_{M}}\right)\Psi^{\|}\left(\frac{\lambda_{g}}{\lambda_{s}\;\Psi \left(\frac{\lambda_{g}}{\lambda_{s}},\frac{\Phi_{m}}{1-\Phi_{M}}\right)},\Phi_{M},\eta \right)\\ \Lambda^{⊥}=\lambda_{s}\;\Psi \left(\frac{\lambda_{g}}{\lambda_{s}},\frac{\Phi_{m}}{1-\Phi_{M}}\right)\Psi^{⊥}\left(\frac{\lambda_{g}}{\lambda_{s}\;\Psi \left(\frac{\lambda_{g}}{\lambda_{s}},\frac{\Phi_{m}}{1-\Phi_{M}}\right)},\Phi_{M},\eta \right) \end{array}$$

Although the transcendental Equations (13) and (15) have no analytical solution, they can easily be solved numerically. Thus, (17) provides an expression of Λ as a function of the five parameters $\lambda_{s}$, $\lambda_{g}$, $\Phi_{m}$, $\Phi_{M}$ and η.

The predictions of (17) are plotted in Figure 11 versus $\lambda_{s}/\lambda_{g}$, for the geometrical parameters of [S1] and [S2]. Their deviations from the results of DNS in the tomographic images are given in Table 3 for various values of $\lambda_{s}/\lambda_{g}$. In spite of large departures which can exceed 10% in the unphysical range of $\lambda_{s}/\lambda_{g}\leq$ 0.1 or ≥ 10${}^{2}$, the agreement in the practical range 1 $\leq \lambda_{s}/\lambda_{g}\leq 20$ is very good. In particular, the most important component $\Lambda^{⊥}$ is predicted within at most 1.6% ([S1]) or 1.1% ([S2]).

It appears that the predictions (17) of DEM are actually even better than those of DNS in reconstructed PEM/PSM samples. A thorough analysis of the contributions to the deviations of the various steps of the modeling was conducted in [19]. Several intermediate procedures have been considered. In a first series, the real macropore geometry extracted from the tomographies was kept, with the mesopores replaced by Poissonian spheres, or the mesoporous material replaced by an equivalent continuum with the conductivity predicted by (16). The same two variants were considered with the macropores replaced by Poissonian ellipsoids. It appears that the best results are obtained when identical procedures are applied for the two scales of homogenization, i.e., either replace both meso and macropores by Poissonian spheres and ellipsoids and operate a DNS in the PEM/PSM samples, or apply a DEM model on both meso and macroscale. The better performance of the latter approach is fortunate, since it is computationally unexpensive, and as seen later, most suited for extension in an evolutive model.

### 4.4. Conductivity of the Apparent Solid

So far, Λ has been determined as a function of the conductivity $\lambda_{s}$ of the apparent solid, which is yet to be determined. Use will be made again of the DEM scheme for this purpose. However, whereas DEM can apply in media where a continuous matrix contains disconnected inclusions, it is inappropriate if both phases are connected and can percolate by themselves. A more adequate model is provided for such situations by the Symmetric Self-Consistent scheme (SSC) [28,30], where N≥ 2 phases are treated symmetrically and can all give rise to continuous paths. In isotropic media, SSC makes use of the solution for the perturbation induced by a sphere in a medium of a different nature and of a self-consistency argument: the perturbations induced by a sphere immersed in a uniform medium with the effective conductivity Λ to be determined should average to zero. This results in

(18) $$\sum_{j=1}^{N}X_{j}\frac{\lambda_{j}-\Lambda }{\lambda_{j}+2\Lambda }\;=\;0$$

An explicit analytical solution when N=2 is possible but (18) must be solved numerically if N≥3. Again, a generalization of SSC exists if the medium is constituted of aligned ellipsoidal elements [8], but it is not required for the present application.

Consider first the mineral grains. In states [S1] and [S2], they are composed of nanoporous alumina. Both phases are connected, the solid because of the grain mechanical integrity and the pores because a continuous path must exist for the escape of the steam released by the dehydration. Thus, the grain conductivity $\lambda_{grain}$ can be evaluated by (18) applied to gas and alumina, with volume fractions $\phi_{n}$ and $1-\phi_{n}$, respectively.

The apparent solid in [S1] is an EVA matrix containing two kinds of fairly spherical inclusions: mineral grains, with conductivity $\lambda_{grain}$ and volume fraction $X_{grain}/\left(1-\Phi_{app}\right)$≈ 0.361, and micropores with conductivity $\lambda_{g}$ and volume fraction $\Phi_{\mu }/\left(1-\Phi_{app}\right)$≈ 0.039. Both can be accounted for by the DEM model. In practice, (13) is applied first for the gas bubbles in EVA. Then, (13) is applied again for the mineral grains in the microporous polymer. The proper order for these two steps is not self-evident, but it was checked that nearly identical results for $\lambda_{s}$ are obtained if the order is reversed.

In state [S2], the polymer has been entirely decomposed. The apparent solid contains only gas and nanoporous alumina grains, with volume fractions $\Phi_{\mu }/\left(1-\Phi_{app}\right)$≈ 0.443 and $X_{grain}/\left(1-\Phi_{app}\right)$≈ 0.557, respectively. The mechanical integrity of the residue tells that the solid phase is connected, and the microporosity has to be connected, so that the pyrolytic gases emitted during the EVA decomposition had a way to escape. Therefore, the conductivity $\lambda_{s}$ of the apparent solid is evaluated by the SSC scheme (18).

**Figure 12:**
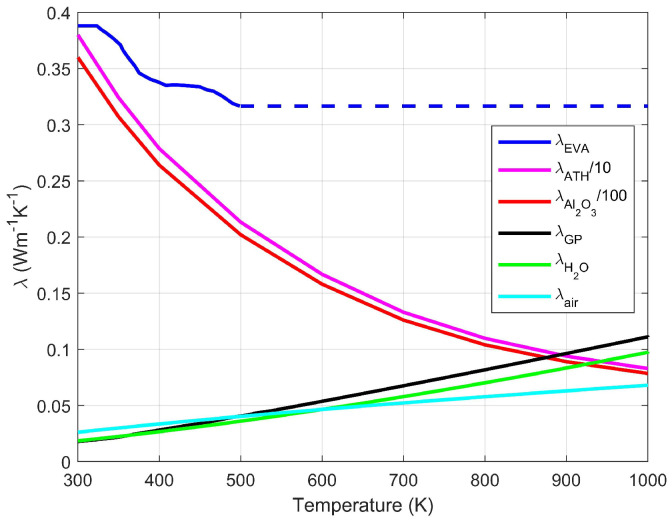
Conductivities of the constituents, as functions of temperature.

In summary, the modeling of the thermal conductivity of [S1] and [S2] is a four-stage upscaling procedure, for the nanoscale (grains), microscale (apparent solid), mesoscale and macroscale (observable in the tomographies). It relies on combinations of DEM and SSC models, according to the morphological features observed on the various scales, and involves six geometrical parameters $\phi_{n}$, $\Phi_{mu}$, $\Phi_{m}$, $\Phi_{M}$, $X_{grain}$ and η which all result from measurements. This has to be supplemented with the conductivities of the constituents, which were taken from [18] for EVA, from [31] for alumina and from [32] for steam and the pyrolytic gases which fill the pores during the successive steps of ATH dehydration and EVA decomposition. Their dependence on temperature is illustrated in Figure 12. The conductivity of ATH at room temperature was extracted from the data of [33], by an inversion procedure described in ([19], App. A.5). Its evolution with temperature was assumed to parallel that for alumina, which is actually very close to the 1/T decay predicted theoretically by [34] for minerals.

### 4.5. Evolutive Conductivity Model

The last remaining task is to extend the conductivity model to describe the material thermal conductivity in states intermediate between [S0], [S1] and [S2], based on the morphological evolution scenario presented in Section 3.2. Ultimately, Λ should be related directly to the state variables available during a numerical simulation, namely, the advancement parameters ($\alpha_{1}$, $\alpha_{2}$) and the temperature *T*.

Recall that the four void fractions $\phi_{n}$, $\Phi_{mu}$, $\Phi_{m}$ and $\Phi_{M}$ are related to $\alpha_{1}$ and $\alpha_{2}$, as described in Section 3.2 and summarized in Figure 9. The intumescence factor is deduced from them by $\beta =1/\left[1-\Phi_{\mu }-\Phi_{m}-\Phi_{M}\right]$ when $\alpha_{2}=0$ and remains constant when ($\alpha_{1}$ = 1, $\alpha_{2}>0$). The grain volume fraction $X_{grain}$ is given by (4). Finally, the shape factor η is given by (10a,10b).

Several parts of the upscaling procedure can be directly applied as they stand in any intermediate state. Specifically, on the meso and macroscale, (17) relates Λ to the current values of the geometrical parameters and to the apparent solid conductivity $\lambda_{s}$ without need for any modification. On the microscale, the structure of the apparent solid does not change between [S0] and [S1]. It consists of mineral grains and an increasing amount of micropores in EVA, and the double use of DEM still applies with the current values of the volume fractions and $\lambda_{grain}$.

However, two steps of the procedure require adaptations. The first one is easy. Before reaching state [S1], the mineral grains contain a remaining amount of ATH. They are therefore a three-phase medium, with ATH, alumina and nanopores. Their volume fractions directly result from $\alpha_{1}$, by (7,8) and the densities of the constituents. We have no direct evidence of their spatial distribution, but as already argued, both the solid and void phases are connected. In addition, ATH and alumina are probably intimately intermixed. Therefore, the SSC model is applied, with *N* = 3 in (18). [S1] with *N* = 2 is only a particular case of this general situation, when the fraction of ATH vanishes. Later, from [S1] to [S2] ($\alpha_{1}$ = 1, $\alpha_{2}>$0), the grain structure does not evolve any more and $\lambda_{grain}$ depends only on *T*, through the temperature dependence of $\lambda_{g}$ and $\lambda_{Al_{2}O_{3}}$.

The other issue is more complex. The apparent solid in [S1] is made of mineral grains and micropores in EVA. Later ($\alpha_{2}>$ 0), the amounts of EVA and micropores decrease and increase, respectively, until only voids and grains remain in [S2]. On the basis of connectivity arguments, DEM was applied in [S1], and SSC in [S2]. Therefore, a change of formulation, and as a matter of fact, two successive changes, have to take place during the evolution between these two states.

The micropore fraction in the EVA ranges from 0.061 in [S1] to 1 in [S2]. The micropores are regarded as inclusions in [S1] and DEM is applied to obtain the conductivity of the microporous EVA. This is obviously inappropriate when the microporosity becomes predominant, and the SSC model is applied instead in the late stages. The switch is made somewhat arbitrarily when the volumes of EVA and micropores are equal. The exact choice of this threshold is actually of little consequence, since the contrast of the conductivities of the gas and EVA is moderate (ratio ∼ 4 at the temperature of pyrolysis), and the jump when switching from DEM to SSC is very small.

The apparent solid also contains mineral grains, which were regarded as separate inclusions in state [S1] and accounted for by the DEM scheme. However, the volume of apparent solid decreases during the EVA decomposition (see Figure 7). Therefore, the grains get more concentrated and they become connected at some point. This is known for a fact in the final state [S2], since the residue is mechanically stable, though very friable. Thus, SSC is more adequate to represent the bicontinuous mixture of grains and gas in the late stages. Again, there is no obvious choice for the moment of the switch from DEM to SSC. The volume fraction of grains in the apparent solid varies from 0.361 in [S1] to 0.557 in [S2], and it is chosen here to switch half-way through this range, i.e., when the grain fraction is 0.459. This corresponds to the solid fraction in a packing of slightly aspherical particles, with sphericity index ∼0.86 [35]. The switch induces a jump in the conductivity of the apparent solid more significant than the change of model for the microporosity, because of the larger conductivity of the solid grains. However, it should be noted that this discontinuity corresponds to an actual percolation transition of the most conducting phase in the material. It is therefore not a spurious effect but an expectable physical feature.

### 4.6. Application of the Model

**Figure 13:**
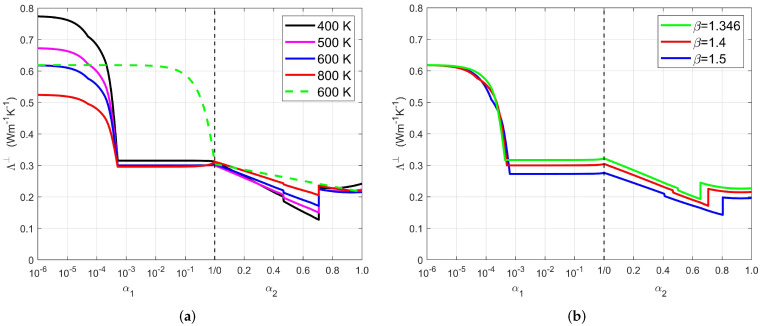
The effective conductivity $\Lambda^{⊥}$ as functions of the ATH dehydration ($\alpha_{1}$) and EVA decomposition ($\alpha_{2}$) advancements, at various temperatures with β = 1.4 (**a**), and at *T* = 600 K with various intumescence factors (**b**). The scale is logarithmic for $\alpha_{1}$ and linear for $\alpha_{2}$. The green broken line in (**a**) corresponds to a linear interpolation between states [S0], [S1] and [S2], when *T* = 600 K.

The effective conductivity $\Lambda^{⊥}$ in the direction of intumescence is shown in Figure 13a as a function of $\alpha_{1}$ and $\alpha_{2}$. In a degradation process during a fire or a cone calorimeter test, ($\alpha_{1}$,$\alpha_{2}$) and *T* evolve jointly but they are not univocally related, unlike in Thermogravimetric Analysis (TGA). Due to the characteristic times for reactive and transport mechanisms, the response depends on the position within the material and on the overall boundary conditions, including the magnitude and history of the incident heat flux. For this reason, the results in Figure 13 are presented as if ($\alpha_{1}$, $\alpha_{2}$) and *T* were independent state variables. The former determines the morphology and composition of the material, and the latter the conductivities of the constituents. Generally, *T* = 500 or 600 K is representative of the conditions during the dehydration step, and *T* = 800 K of those during the EVA decomposition. However, less typical situations are possible due to changes of the outer conditions. Large temperatures can occur in the first stage because of a strong and sudden increment of the incident radiative flux, and conversely, *T* can culminate and decrease if the fire and the thermal flux subside.

Several events can be observed during the evolution of $\Lambda^{⊥}$, which correspond to the onset of new morphological features, occurring at the advancements listed in Table 2. Note that in order to distinguish some very early events with important consequences, a logarithmic scale is used for $\alpha_{1}$.

A first drop of $\Lambda^{⊥}$ by about 10% occurs in the interval 0 ≤ $\alpha_{1}$ ≤ $\alpha_{1\mu }$ due to the apparition of the microporosity. Then, a second and more important decrease results from the successive developments of the meso and macropores, for $\alpha_{1\mu }$ ≤ $\alpha_{1}$ ≤ $\alpha_{1M}$, without visible accident in the curves at the transition $\alpha_{1m}$ between these two steps. At this point, intumescence is fully developed. The only change during the subsequent period until $\alpha_{1}$ = 1 is the increase of the nanoporosity due to the dehydration of the remaining ATH. However, ATH is converted into alumina and steam, with conductivities larger and smaller than $\lambda_{ATH}$, respectively, and it turns out that the effects nearly compensate. Thus, $\lambda_{grain}$, and in turn, $\lambda_{s}$ and $\Lambda^{⊥}$ remain quasi-constant in this interval. When dehydration is complete ($\alpha_{1}$ = 1), $\Lambda^{⊥}$ is about 30% of its initial value at room temperature.

During the second phase ($\alpha_{1}$ = 1, $\alpha_{2}$> 0), the micro, meso and macroporosity increase successively which causes a further decrease of $\Lambda^{⊥}$. Nothing particular is observed when $\alpha_{2}$ = $\alpha_{2\mu }$ or $\alpha_{2m}$, at the transitions between these three steps. However, two discontinuities are seen, induced by the changes of upscaling models mentioned in Section 4.5, from DEM to SSC. The first one corresponds to the transition to percolation of the microporosity. At the typical temperature 800 K for this stage of the process, it is of a very small amplitude since the contrast of the conductivities of the gas and of the embedding EVA is moderate and the choice of upscaling model makes little difference. It is more significant when *T* = 400 K, because $\lambda_{g}$ is much smaller (see Figure 12) and the conductivity contrast is much larger. The second discontinuity corresponds to the transition to percolation of the mineral grains and its amplitude is more important, especially at low temperatures because the ratio of the conductivities of the grains and of the embedding gas is larger, as already mentioned. Recall however that a physical transition does indeed take place, and therefore, it has to echo in the conductivity evolution. Its manifestation might be less sharp and not at the precise value of $\alpha_{2}$ resulting from our choice, but it has to take place somewhere near it.

The final part of the curves shows a positive inflection and even an increase of $\Lambda^{⊥}$ when *T* = 400 K. This is because alumina, the most conducting constituent, is arranged in connected grains and its conductivity strongly increases when *T* decreases.

It is interesting to compare the predictions of the present model with those of the most common approach in the literature (see e.g., [36]). Assuming that the conductivities are known for the material in a few characteristic states, such as [S0], [S1] and [S2] here which correspond to the completion of successive chemical reactions, they are generally combined by a mixing rule to deduce the effective conductivity of the material from its current composition (or equivalently from chemical advancement parameters) in any stage of its degradation. As an illustration, the result of such a procedure is shown in Figure 13a (green broken line), for *T* = 600 K. Dramatic differences are observed, because the simple interpolation does not take into account the changes in the microstructure which can result from the constituent degradation. The volume average is always an upper bound for the conductivity of a mixture, and it can be a gross overestimate depending on geometrical arrangement of the various phases. This occurs here in the early stage during the onset of the intumescence, and in lesser respect during the decomposition of EVA.

## 5. Discussion

### 5.1. Sensitivity to Some Possible Sources of Error

We are now in a position to justify a posteriori the hypothesis whereby radiative transfers contribute only marginally to heat propagation in the material. Consider the idealized picture of a periodic stratified medium, inspired by Figure 3a or Figure 8, containing void layers with conductivity $\lambda_{g}$, thickness $b_{g}$ and volume fraction $\Phi_{app}$, intertwined with slabs of the apparent solid with conductivity $\lambda_{s}$. This maximizes the relative importance of radiative exchanges through the gaseous layers, since this structure causes continuous barriers of the least conducting phase to hinder the heat flux in the intumescence direction. Indeed, $\Lambda^{⊥}$ for this model is equal to the harmonic average ${\langle \lambda \rangle }_{H}$ of $\lambda_{g}$ and $\lambda_{s}$, which is only about 2/3 of the values obtained in Section 4.6. It is an easy matter to account for the emissions by the solid surfaces regarded as gray bodies. After linearization of the jumps of $T^{4}$, radiation contributes to heat transfer in the form of a Rosseland radiative conductivity $\Lambda_{r}^{⊥}$, given to leading order by

(19) $$\Lambda_{r}^{⊥}\approx 4\epsilon \sigma {\bar{T}}^{3}\;b_{g}\Phi_{app}{\left(\frac{{\langle \lambda \rangle }_{H}}{\lambda_{g}}\right)}^{2}$$

where ϵ it the emissivity, σ is the Stefan–Boltzmann constant and $\bar{T}$ is the typical temperature. The thickness $b_{g}$ can be deduced from the volumetric area, which results via (2) from the correlation measured in the direction normal to the strata plane. Applications of (19) with ϵ = 0.9 and the parameters for [S1] at $\bar{T}$ = 610 K (peak of the mass loss rate due to dehydration in TGA tests), or with those for [S2] at $\bar{T}$ = 730 K (peak of EVA decomposition) yield nearly identical values $\Lambda_{r}^{⊥}\approx$ 0.008 Wm${}^{-1}$K${}^{-1}$. Though not entirely negligible, this is only a few percent of the values of $\Lambda^{⊥}$ in Figure 13.

As already mentioned in Section 2.3, the measurement of the intumescent factor β is not highly accurate. Its value plays no role in the determination of the characteristics of the meso and macroscopic features visible in the tomographies (volume fractions, correlations, pore size spectrum, anisotropy), but it directly influences the estimates of the grain volume fraction $X_{grain}$ via (4) and of the microporosity via (5). An illustration of the consequences of a misevaluation of the intumescence is provided in Figure 13b. Two values of β are considered in addition to the measured value 1.4. One is the lower limit β = 1.346; in view of $\Phi_{app}$ observed in [S1], the sample volume has necessarily increased by at least this ratio. The other one β = 1.5 is a very conservative upper bound. It is unlikely that the actual intumescence deviates that much from the measurement 1.4. Aside from slight shifts in the changes of regimes (or disappearance, since $\Phi_{\mu }^{\left(1\right)}$ = 0 when β = 1.346), the three curves have identical aspects. They are shifted vertically, mostly because of the different microporosities. Conductivity is seen to decrease when intumescence increases, which is the very idea of using intumescent materials for fire protection purposes. Variations of β by ±0.05 induce changes of $\Lambda^{⊥}$ by about ∓5% when 0 $<\alpha_{1}<$1 and ∓4% when 0 $<\alpha_{2}\leq$1.

The consequences of other modeling choices or sources of uncertainty have been examined in (Section 7.3 for $\lambda_{s}$ and 8.4.3 for $\Lambda^{⊥}$ [19]), including the choice of the thresholds for the switches from DEM to SSC models, and misevaluations of the initial amount of ATH in the material. This is not detailed here for brevity. Let us just mention that a 60% mass fraction of ATH is near the lower end of the practical range for flame retarded EVA/ATH mixtures. The whole modeling procedure was repeated, keeping all the geometrical observations but assuming a 65% mass fraction of ATH, near the upper end of the practical range. Several effects are observed. First, since a greater overall amount of steam is to be released, the thresholds $\alpha_{1\mu }$ to $\alpha_{1M}$ in Table 2 are smaller by a factor 60/65. Secondly, since the volume fraction $X_{grain}$ of the grains is larger, their transition to percolation and the associated switch from DEM to SSC upscaling occur sooner, for $\alpha_{2}$≈ 0.55 vs. ≈0.71 in Figure 13. However, most importantly, the larger amount of the very conducting alumina causes $\Lambda^{⊥}$ to increase, by about 5% and 14% in states [S1] and [S2], respectively.

### 5.2. Confrontation with Experiments

Most commonly, the validation of numerical models of thermal degradation relies on the comparison of their response to that of experiments, such as cone calorimeter tests, in terms of global parameters (e.g., mass loss rate or heat release rate) [2,3,4,5,37]. This is ill-suited for the specific assessment of the performances of our sub-model relating the effective conductivity to chemical advancement parameters. First, the simulations can involve many other sources of errors, including flaws in the accounts of transport and thermochemical mechanisms in the model itself, and uncertainties in the boundary conditions to be applied to represent the test conditions. Furthermore, the temperature and the state of degradation in these experiments are generally not spatially uniform. Indeed, due to limiting transport processes, thermal and reaction fronts generally propagate from the exposed face into the depth of the material, as illustrated, for instance, in Figure 15 of [38] and in Figures 2 and 3 of [39]. Therefore, the global response convolves the profile of $\Lambda (\alpha_{1},\alpha_{2},T)$, which makes it very difficult to pinpoint errors in its modeling.

Obtaining samples of materials in a well characterized state of degradation and then measuring the thermal conductivity of these fragile objects for a direct check of the prediction of $\Lambda (\alpha_{1},\alpha_{2},T)$ is a difficult task, but fortunately, a set of measurements in conditions approaching these requirements was implemented in [17,18]. The material was an EVA/ATH mixture similar to that considered here, although it contained 65%m of filler and we cannot be sure that the polymer formulation is exactly identical. The conductivity was measured by the transient plane source (or hot-disc) method with two 25 × 25 × 7 mm${}^{3}$ slabs placed on either side of the probe. Considering the slab’s aspect ratio, we assume that the measured conductivity corresponds to $\Lambda^{⊥}$ along the direction normal to both the sensor and the wide faces exposed to the heat source.

**Figure 14:**
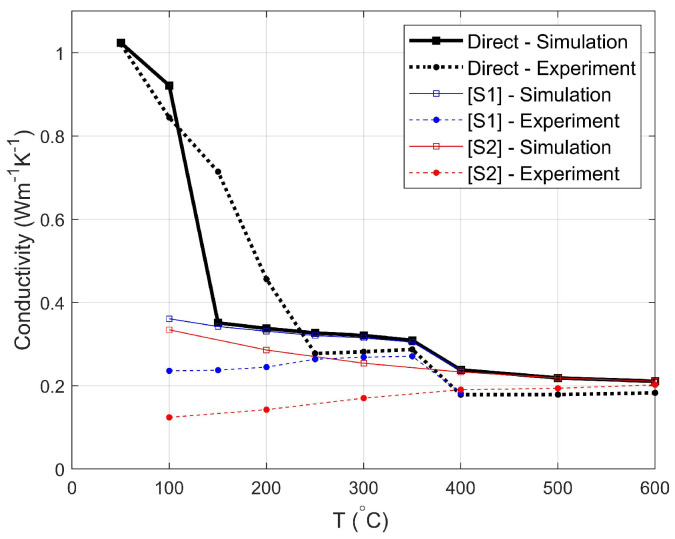
Effective conductivity measurements of [17,18], and corresponding results of simulations coupling the conductivity model with the pyrolysis simulation tool CALIF${}^{3}$S—ISIS.

In a first (so-called “direct”) experiment, this assembly was put in a furnace and submitted to successive 50 ${}^{{}^{\circ}}$C temperature increments. The conductivity measured after each of these 150 min isothermal steps is plotted as a function of *T* in Figure 14 (dotted black curve). It should be noted that they can be compared to the model $\Lambda^{⊥}\left(\alpha_{1},\alpha_{2},T\right)$ only because a mapping from *T* to ($\alpha_{1}$,$\alpha_{2}$) is possible, considering the scenario. A priori, decomposition is not started ($\alpha_{1}$ = $\alpha_{2}$ = 0) when *T* ≲ 200 ${}^{{}^{\circ}}$C, and only the temperature dependence of the constituents plays a role. *T* ≈ 350 ${}^{{}^{\circ}}$C corresponds to complete dehydration, i.e., state [S1] ($\alpha_{1}$ = 1,$\alpha_{2}$ = 0), and *T* ≳ 500 ${}^{{}^{\circ}}$C to the final state [S2] ($\alpha_{1}$ = $\alpha_{2}$ = 1).

Our model for the conductivity was coupled with the pyrolysis simulation tool CALIF${}^{3}$S-ISIS [5,19], and 1D simulations were conducted reproducing the experimental set-up and heating history. The required physical parameters, such as heat capacities and emissivity, and the coefficients of the Arrhenius kinetics laws were taken from [17,18]. The conductivity $\Lambda^{⊥}$ observed after each 150 min step at the sensor position is shown in Figure 14 (solid black curve).

The DEM prediction for the initial conductivity is in excellent agreement with the measurement. Note that it is larger than in Figure 13 because of the 65% (vs. 60%) mass fraction of ATH. Then, a sharp drop takes place, because the conductivities of the initial constituents are decreasing functions of *T*, but also because of the development of the multiscale porosity. A first plateau is reached, both numerically and experimentally. This is consistent with Figure 13, where $\Lambda^{⊥}$ is nearly constant during most of the ATH dehydration, once intumescence is established ($\alpha_{1M}\leq \alpha_{1}\leq 1$). The numerical and experimental values are in a good 7% agreement when the material is in state [S1].

A second drop, which corresponds to the gradual decrease in Figure 13 during EVA decomposition ($0\leq \alpha_{2}\leq 1$), occurs mostly in the range 350 to 400 ${}^{{}^{\circ}}$C, followed by a second plateau. Beyond 500 ${}^{{}^{\circ}}$C, the material is in state [S2] and the model exceeds the measured value by about 16% (0.03 to 0.04 Wm${}^{-1}$K${}^{-1}$). This agreement is fair, but not outstanding. In state [S2], the model involves three successive upscalings, for the conductivity of the nanoporous alumina grains, for that of the apparent solid, and that of the meso and macroporous apparent solid. There might be room for improvement if a better knowledge of the structure of the apparent solid (grains and micropores) were available, from tomographies with a finer resolution. However, it should also be noted that in this very porous final state, $\Lambda^{⊥}$ is very sensitive to the nature of the pore filling gas. At this stage, it is generally pyrolytic gases (λ≈ 0.09 Wm${}^{-1}$K${}^{-1}$), as assumed in Figure 13, but in this experiment, after a 150 min rest period, it is probably the gas in the atmosphere surrounding the sample (nitrogen, or nearly equivalently air, λ≈ 0.06 Wm${}^{-1}$K${}^{-1}$). The latter was assumed in the simulations.

The most salient difference between the curves for the modeled and measured $\Lambda^{⊥}$ is in the shape of the initial drop in the range 100 ${}^{{}^{\circ}}$C ≤T≤ 200 ${}^{{}^{\circ}}$C. In the simulations, it results primarily from the development of the porosity and the resulting intumescence, because ATH dehydration is already initiated, in spite of the low temperature. Regarding the measurements, the statement in [17,18] that “The pristine EVA/ATH does not decompose before the isothermal step 200 ${}^{{}^{\circ}}$C” suggests that the decay of $\Lambda^{⊥}$ results only from that of $\lambda_{EVA}$ and $\lambda_{ATH}$. This seems questionable, first because the decrease of $\lambda_{EVA}$ and $\lambda_{ATH}$ (Figure 12) is not sufficient to explain the drop of $\Lambda^{⊥}$, but also because the quoted statement applies to TGA tests conducted with heating rates faster than in the present experiment.

Even a low kinetic reaction rate can result in a non-negligible advancement if maintained for a long time, and recall that the dehydration of only a small fraction of ATH is needed to create the volume of steam corresponding to the porosity (see Table 2). With a heating rate of 2K/min, a noticeable mass loss already occurs below 200 ${}^{{}^{\circ}}$C ([18], Figure 52) and the 50 ${}^{{}^{\circ}}$C/150 min steps in the present experiment are a still much lower rate. Thus, we believe that the drop of the measured $\Lambda^{⊥}$ also involves a partial dehydration of ATH. It remains that its onset and its effect on the conductivity is too sharp in the simulations. This can only result from the parameters of the Arrhenius kinetic law used in the simulations (taken from [18]). They have probably been optimized without putting specific weight on the small early mass loss, and based on TGA data with faster heating rates than in this experiment. However, since these faster rates are more representative of the targeted situation of the material exposed to a fire, the kinetic parameters are probably appropriate for practical applications, even if they induce discrepancies in the early part of Figure 14.

A few other features should be kept in mind when comparing the model response to the experimental data. On one hand, the simulations were conducted in a 1D formulation. Since the aspect ratio of the slabs is only 25/7, 3D effects cannot be entirely ruled out, especially since the material structure and conductivity are anisotropic. On the other hand, whereas it was checked in the simulations that *T* is uniform in the samples at the end of each 150 min period, when conductivity is measured, it is not so right after the temperature increments are imposed. During these transient episodes, *T* is larger near the sample outer boundaries, and in turn, degradation progresses faster than near the centre, where the sensor is located.

The advancement parameters $\alpha_{1}$ and $\alpha_{2}$ at the instants of the measurements are given in Table 4, as obtained in the simulations, at the centre and outer boundary of the sample. Note first that at T≤ 200 ${}^{{}^{\circ}}$C and at *T* = 350 ${}^{{}^{\circ}}$C, where the material is reputedly in its [S0] and [S1] states, respectively, ATH dehydration and EVA decomposition are already initiated. Secondly, $\alpha_{1}$ at *T* = 250 ${}^{{}^{\circ}}$C is noticeably different in the inner and outer regions of the sample; the same applies for $\alpha_{2}$ at *T* = 400 ${}^{{}^{\circ}}$C. Thus, even in this particularly careful experiment, the aforementioned mapping from *T* to ($\alpha_{1}$,$\alpha_{2}$) is still slightly equivocal.

In a second set of experiments (labeled here “S1” and “S2”), pre-degraded samples were prepared by submitting them to *T* = 350 or 500 ${}^{{}^{\circ}}$C during 150 min. This brings them admittedly to states [S1] and [S2]. After their cooling, successive steps of 50 ${}^{{}^{\circ}}$C (S1) of 100 ${}^{{}^{\circ}}$C (S2) were applied and the conductivity was measured. Thus, this very nice set of experiments aimed to evaluate as a function of *T* the conductivity of the material in states [S0] (“direct”, for *T* ≲ 200 ${}^{{}^{\circ}}$C), [S1] (S1, for *T* ≲ 350 ${}^{{}^{\circ}}$C) and [S2] (S2, for *T* up to 700 ${}^{{}^{\circ}}$C). The results are shown in Figure 14. They are close to those of the direct experiment in the ranges of *T* where [S1] and [S2] prevail. The small differences give an idea of the good accuracy and repeatability of the measurements: ≈6% between “direct” and S1 at *T* = 350 ${}^{{}^{\circ}}$C and ≈9% between “direct” and S2 at *T* = 500 ${}^{{}^{\circ}}$C. The results of simulations for the alternative scenarios are also shown in Figure 14 and they are also very close to those for the “direct” simulations in the temperature intervals where [S1] and [S2] prevail. Thus, there is little to add to the discussion, except for the following observation: the experimental and numerical determinations of $\Lambda^{⊥}$ in the pre-degraded samples strongly deviate at low temperatures. In particular, $\Lambda^{⊥}$ is found numerically to decrease with *T*, whereas its measured values increase. This is a perplexing feature. $\Lambda^{⊥}$ is found much larger than the gas conductivity, both numerically and experimentally, which means that the solid contribution dominates (EVA in and alumina [S1] and alumina in [S2]). However, both $\lambda_{Al_{2}O_{3}}$ and $\lambda_{EVA}$ decrease with *T* (Figure 12). We have no explanation for the opposite trend of $\Lambda^{⊥}$ observed in the experiments.

## 6. Concluding Remarks

An evolutive conceptual model has been proposed for the morphology and heat conductivity of the intumescent EVA-ATH composite during its thermal degradation. It accounts for the multiscale and anisotropic structure observed during the analysis of tomographic images of samples at representative stages of degradation. The conductivity is directly related to the geometrical characteristics and in turn to temperature and chemical advancement parameters by analytic expressions, which is particularly convenient for an implementation in a pyrolysis simulation tool. Furthermore, the expressions result from rationalized geometrical considerations, and in turn, from the composite initial composition. Therefore, the model presents a degree of versatility. It remains applicable to mere changes of input parameters if the composite formulation or its constituent properties are modified moderately enough so that the overall fire behavior is not qualitatively changed. For instance, it was applied in [19] with different initial weight fractions of ATH.

As it stands, the model satisfactorily reproduces the conductivity as obtained by direct numerical simulations in the geometries of particular states available from tomographic images, and experimental measurements conducted with a similar EVA-ATH composite.

On the same geometrical basis, it is conceivable to try and model other effective transport coefficients, pertaining for instance, to gas flow or gaseous species diffusion. However, since these transfer take place exclusively in the pores, unlike heat conduction, which involves both the pore and (often primarily) solid domains, more attention should be paid to the connectivity of the gaseous inclusions. For this purpose, tomographies with a better spatial resolution are required.

More generally, more and better tomographic images are highly desirable for several reasons. On one hand, imaging the material in degradation states intermediate between [S0], [S1] and [S2] could bring support to the scenario for the morphological evolution, which so far relies on reasoned but partly conjectural arguments, or possibly reveal inadequacies and provide directions for its amendment. On the other hand, a finer resolution could allow one to pinpoint more accurately the transitions to percolation of the micropores and of the solid grains involved in the conductivity modeling (Section 4.5). These transitions have to occur, but for the time being, the instant when they do is set partly arbitrarily.

Improved resolution would also eliminate the distinction between micro and mesopores, which is only an artifact of metrological limitations, or might reduce the fraction of unseen porosity sufficiently to make it negligible. A complete vision of the pore size spectrum would be helpful if the continuous size distribution observed in Appendix A.1 were to be used instead of the current trimodal schematization in micro, meso and macropores. Such an extension of the model would be conceptually gratifying, but it is not certain that it would significantly modify the conductivity predictions. The trimodal model seems to be a smooth enough approximation, since no discontinuity nor change in slope is visible in Figure 13 at the transitions between the developments of micro and mesoporosity ($\alpha_{1\mu }$ and $\alpha_{2\mu }$) or between the developments of meso and macroporosity ($\alpha_{1m}$ and $\alpha_{2m}$). The only accidents are due to physical events (completion of the intumescence or percolation transitions). The same applies for other simplifying features of the model, such as the strict alignment of the anisotropic macropores. Ellipsoids with orientations slightly wobbling around a preferential direction can be accounted for straightforwardly by the DEM scheme, but it would result only in a second-order correction to (17). Thus, the priority in future studies should be less so the refinement of technical details than the validation and possibly improvement of the modeling concepts.

It should be noted that beyond their application in the present work, the methodology and conceptual tools implemented here can be of interest by themselves for the treatment of other materials and in other contexts. This applies to the global modeling approach, and to some technical aspects which are involved. In particular, the method for the characterization of the gaseous inclusions shape (Appendix A) is original. The tensorial distance (Appendix B) used to assess the quality of the conductivity predictions is also a new contribution, mentioned in the literature only in the recent related works [26,40].

Finally, radiative exchanges, which have here a minor but not entirely negligible contribution to heat transfers, are an obvious subject for future work. The other most common cable sheath material is PVC. It is also intumescent, and therefore it also gives rise to gaseous inclusions in the polymer matrix. However, much larger cavities develop, and as a result, radiative heat transfers (unaccounted for in the present work) play a dominant role, as shown in the preliminary study [37]. Just like conduction, they could be addressed by direct simulations in the tomographic images of the samples, or by a more theoretical approach in the object-based conceptual model of the geometry. Therefore, both the geometric modeling and the accounting for radiative transfers call for additional developments. 

## Figures and Tables

**Table 1:** Measured porosities in [S1] and [S2], and geometrical parameters for the PSM/PEM model.

	Apparent Porosity	Meso-Porosity		Macro-Porosity			
	$\Phi_{\mathrm{app}}$	$\Phi_{m}$	R	$\Phi_{M}$	η	A	B = C
[S1]	0.257	0.072	30 μm	0.185	5.5	70 μm	385 μm
[S2]	0.519	0.178	30 μm	0.341	2.9	47 μm	136 μm

**Table 2:** Thresholds of the advancements $\alpha_{1}$ and $\alpha_{2}$ for porosity evolution.

	ATH Dehydration	EVA Decomposition
Microporosity	$\alpha_{1\mu }$ = 5 × 10${}^{-5}$	$\alpha_{2\mu }$ = 0.413
Mesoporosity	$\alpha_{1m}$ = 1.8 ×10${}^{-4}$	$\alpha_{2m}$ = 0.652
Macroporosity	$\alpha_{1M}$ = 5.1 ×10${}^{-4}$	$\alpha_{2M}$ = 1

**Table 3:** Comparison of the conductivity tensors resulting from DNS in the PEM/PSM model (left) of from the analytical DEM model (right) with those obtained by DNS in the tomographic images, in terms of the relative deviations of $\Lambda^{⊥}$ and $\Lambda^{\|}$ and of the tensorial distance $D^{\prime }$. Data in boldface are the maximal deviations in the practical range of the most important component quantity $\Lambda^{⊥}$.

	PEM/PSM vs. Tomographies				Analytic Model vs. Tomographies			
$\lambda_{s}/\lambda_{g}$	**0.1**	**20**	**10${}^{2}$**	**10${}^{3}$**	**0.1**	**20**	**10${}^{2}$**	**10${}^{3}$**
[S1], $\Lambda_{PEM}^{⊥}/\Lambda_{Tomo}^{⊥}-1$	0.005	**0.040**	0.074	0.087	−0.073	**−0.016**	−0.057	−0.083
[S1], $\Lambda_{PEM}^{\|}/\Lambda_{Tomo}^{\|}-1$	−0.030	0.017	0.031	0.037	−0.066	0.021	0.037	0.043
[S1], $D^{\prime }\left(\Lambda_{PEM},\Lambda_{Tomo}\right)$	0.035	0.027	0.043	0.048	0.079	0.024	0.045	0.052
[S2], $\Lambda_{PEM}^{⊥}/\Lambda_{Tomo}^{⊥}-1$	0.020	**0.004**	0.028	0.041	−0.179	**−0.011**	−0.016	−0.020
[S2], $\Lambda_{PEM}^{\|}/\Lambda_{Tomo}^{\|}-1$	−0.001	−0.013	−0.015	−0.012	−0.103	0.025	0.079	0.107
[S2], $D^{\prime }(\Lambda_{PEM},\Lambda_{Tomo)}$	0.017	0.014	0.018	0.024	0.151	0.028	0.092	0.124

**Table 4:** Advancements $\alpha_{1}$ and $\alpha_{2}$ when $\Lambda^{⊥}$ was recorded in the “direct” simulation, at the sensor position and at the outer boundaries of the sample.

*T* (${}^{{}^{\circ}}$C)	50	100	150	200	250	300	350	400	500	600
$\alpha_{1}$ (sensor)	0	0	0.0011	0.117	0.931	1	1	1	1	1
$\alpha_{1}$ (boundaries)	0	0	0.0011	0.130	0.996	1	1	1	1	1
$\alpha_{2}$ (sensor)	0	0	0	0	0	0.0004	0.036	0.759	1	1
$\alpha_{2}$ (boundaries)	0	0	0	0	0	0.0004	0.037	0.832	1	1

## Abbreviations

The following abbreviations are used in this manuscript:

ATH	Alumina Trihydrate
EVA	Ethylvinyl Acetate
SEM	Scanning Electron Microscopy
TGA	Thermogravimetric Analysis
DNS	Direct Numerical Simulation
PEM	Penetrable Ellipsoid Model
PSM	Penetrable Sphere Model
DEM	Differential Effective-Medium model
SSC	Symmetric Self-Consistent Scheme

## Appendix A. Sizes and Shapes of the Individual Inclusions

### Appendix A.1. Measurements

The geometrical characterization of the inclusions requires first the segmentation of the digital images by the identification of individual pores. This was performed by use of an in-house tool PERCOLF [41]. The individual pores are then characterized by their volumes and shapes.

The measurement of the inclusion volumes is straightforward, and their distribution is shown in Figure A1a. As already noted, the distribution is not bimodal. Instead, it roughly follows a power law $\propto V^{-1.55}$ over a range of volume 1 ≤V≲ 10${}^{3}$ voxels. Larger inclusions are too rare to assess whether this behavior extends any further. Two events on the far right correspond to large fully connected clusters. Since the macropores are at least 2$R_{c,s}$ = 6 voxels thick, their volumes are at least 113 and larger than 200 for most of them.

The inclusion shapes are characterized by measuring their moment of inertia I relative to their barycenter $r_{g}$,

(A1) $$I=\frac{1}{V}\;\int_{V}\left(r-r_{g}\right)\left(r-r_{g}\right)dV$$

and determining its eigenvalues and eigendirections. For an ellipsoid with semi-axes A≤B≤C, the eigenvalues are

(A2) $$I_{\alpha \alpha }=A^{2}/5,\;I_{\beta \beta }=B^{2}/5,\;I_{\gamma \gamma }=C^{2}/5$$

Therefore, the inclusion moments of inertia are interpreted by reference to an equivalent ellipsoid, which is indeed the general visual appearance of the macropores (see Figure 8), with minor (*A*), mid (*B*) and major (*C*) semi-axes deduced from (A2). The eigendirections corresponding to the minor axis are clearly clustered around a preferential direction which corresponds to most rapidly decreasing correlations. For instance, the equivalent ellipsoids of the macropores in [S1] are oblate with their minor axis close to the *z*-direction. The mid and major axes are found in the orthogonal plane, without a clear organization.

As expected, the three semi-axes of the mesopores are found to be very similar, with fluctuations due to their irregular shape but without orientational organization. The semi-axes measured in the macropores are shown in Figure A1b,c as functions of the inclusion volume. They increase of course with the volume, but according to different power laws. In spite of a strong dispersion of the data, a clear general trend exists. Thus, the inclusion anisotropy increases with their size. Starting from nearly spherical for volumes V≲113 (volume of the sphere with a radius equal to the chosen threshold $R_{c,s}$ for the separation of meso and macroporosity), the shape of larger inclusions become more and more oblate, with the ratios B/A and C/A increasing roughly as $V^{0.3}$.

### Appendix A.2. Modeling

There is no technical obstacle to incorporate all the features observed in Figure A1 when generating numerical samples according to the PEM/PSM model described in Section 3.1, and subsequently performing direct numerical simulations as done in Section 4.2. However, continuous size and shape spectra make the application of the theoretical upscaling arguments in Section 4.3 much more complicated. For this reason, the data are schematized in the most simple way, whereby all the individual mesopores are represented by identical spheres with a radius *R* and the all the individual macropores by identical and parallel oblate ellipsoids. The validity of this simplification is of course subject to a posteriori verification that the resulting conductivity predictions are satisfactory. This was checked in Section 4.2 and Section 4.3 and the agreement with the results of DNS in the actual tomographic images is demonstrated in Table 3. The supporting arguments for the simplifications are as follows.

The representation of the meso-inclusions by spheres is supported by various observations such as the isotropy of the correlations measured on the mesopores, and the convergence of the fitting lines in Figure A1b,c. The spectrum of $R_{c}$ in mesopores spans a range of 10 to 30 μm, but whereas small inclusions are more numerous, the largest ones largely dominate in volume (the range 27 to 30 μm contains 50% of the total mesoporosity). Therefore, all spheres in the PSM model are given a same radius *R* equal to the upper value 30 μm. It should be noted that the conductivity predictions are actually independent of the value of *R*, as long as the spheres are much smaller than the ellipsoids (see Section 4.3).

The macro-inclusions are represented by parallel, monodisperse, biaxial oblate ellipsoids with semi-axes A×ηA×ηA, (η>1). Both the alignment and the biaxial character of the ellipsoids stem from observations and from a priori arguments. Note first that the set-up for the sample preparation is axially symmetric. The crucible is cylindrical, and intumescence can only develop along its axis. There is no reason a priori for anisotropy to develop in the transverse plane, except possibly for artifacts in the sample preparation or heating. It is unwise to include such incidental features in a general purpose model. The hypothesis of ellipsoids with identical orientations is supported by the visual observation of Figure 3a, and by the clear preferential direction of the eigendirections associated with the minor moments of inertia $I_{\alpha \alpha }$ of the inclusions. Its moderate random scatter could conceivably be included in the model, but this has not been done here. The biaxial character is also supported in an average sense by the measurements of the inclusion mid and major moments of inertia, $I_{\beta \beta }$ and $I_{\gamma \gamma }$. They can indeed differ for individual inclusions (though both are always clearly larger than $I_{\alpha \alpha }$) as shown in Figure A1b,c, but without orientational organization.

The ellipsoid volume $V_{E}$ is set equal to the volume-weighted average of the measured macro-inclusion volumes $V_{j}$, i.e., to the mean volume of the inclusion containing a point randomly located in the macroporosity. Similarly, η is taken as equal to the volume-weighted average of the measured aspect ratios $\left(B_{j}+C_{j}\right)/2A_{j}$, and the minor semi-axis *A* is set accordingly,

(A3) $$V_{E}=\frac{\sum_{j}V_{j}^{2}}{\sum_{j}V_{j}}\;,\;\eta \;=\;\frac{\sum_{j}V_{j}\frac{B_{j}\;+\;C_{j}}{2A_{j}}}{\sum_{j}V_{j}}\;,\;A\;=\;{\left[\frac{3V_{E}}{4\pi \eta^{2}}\right]}^{1/3}$$

## Appendix B. Tensorial Distance D

The difference between two conductivity tensors $\Lambda_{1}$ and $\Lambda_{2}$, resulting, for instance, from different upscaling protocols, can be quantified by use of the dimensionless distance $D^{\prime }$ defined as follows. In response to a same unit gradient g, these tensors predict fluxes $q_{i}=-\Lambda_{i}\cdot g$. The squared norm of their deviation $\|q_{2}-q_{1}{\|}^{2}$ is equal to $g^{t}\cdot {\left(\Lambda_{2}-\Lambda_{1}\right)}^{t}\cdot \left(\Lambda_{2}-\Lambda_{1}\right)\cdot g$. It is maximum when g is aligned with the eigendirection of ${\left(\Lambda_{2}-\Lambda_{1}\right)}^{t}\cdot \left(\Lambda_{2}-\Lambda_{1}\right)$ associated with its largest eigenvalue. Therefore, we measure the difference of $\Lambda_{1}$ and $\Lambda_{2}$ by the distance D and its normalized dimensionless counterpart $D^{\prime }$ defined by

(A4) $$D^{2}\left(\Lambda_{1},\Lambda_{2}\right)=\mathrm{largest}\mathrm{eigenvalue}\mathrm{of}\;\left[{\left(\Lambda_{2}-\Lambda_{1}\right)}^{t}\cdot \left(\Lambda_{2}-\Lambda_{1}\right)\right]\;,\;D^{\prime }=\frac{D}{{\left[\;\bar{\Lambda_{1}}\;\bar{\Lambda_{2}}\;\right]}^{1/2}}$$

Note that D is a distance in the mathematical sense, i.e., a symmetric positive-definite function satisfying the triangle inequality. It was introduced in [26,40] where its properties are detailed. $D^{\prime }\left(\Lambda_{1},\Lambda_{2}\right)$ is the maximal relative deviation (combining magnitude and direction differences) of the fluxes predicted by $\Lambda_{1}$ and $\Lambda_{2}$ when applied to the same gradient.
